# PGC-1a mediated mitochondrial biogenesis promotes recovery and survival of neuronal cells from cellular degeneration

**DOI:** 10.1038/s41420-024-01953-0

**Published:** 2024-04-17

**Authors:** Wenting You, Kèvin Knoops, Tos T. J. M. Berendschot, Birke J. Benedikter, Carroll A. B. Webers, Chris P. M. Reutelingsperger, Theo G. M. F. Gorgels

**Affiliations:** 1 University Eye Clinic Maastricht UMC+, Maastricht University Medical Center+, Maastricht, The Netherlands; 2 Department of Biochemistry, CARIM School for Cardiovascular Disease, Maastricht University, Maastricht, The Netherlands; 3 Department of Mental Health and Neuroscience, Maastricht University, Maastricht, The Netherlands; 4 The Microscopy CORE lab, Maastricht Multimodal Molecular Imaging Institute, Maastricht University, Maastricht, The Netherlands

**Keywords:** Cellular neuroscience, Mechanisms of disease

## Abstract

Neurodegenerative disorders are characterized by the progressive loss of structure and function of neurons, often including the death of the neuron. Previously, we reported that, by removing the cell death stimulus, dying/injured neurons could survive and recover from the process of regulated cell death, even if the cells already displayed various signs of cellular damage. Now we investigated the role of mitochondrial dynamics (fission/fusion, biogenesis, mitophagy) in both degeneration and in recovery of neuronal cells. In neuronal PC12 cells, exposure to ethanol (EtOH) induced massive neurite loss along with widespread mitochondrial fragmentation, mitochondrial membrane potential loss, reduced ATP production, and decreased total mitochondrial volume. By removing EtOH timely all these mitochondrial parameters recovered to normal levels. Meanwhile, cells regrew neurites and survived. Study of the mitochondrial dynamics showed that autophagy was activated only during the cellular degeneration phase (EtOH treatment) but not in the recovery phase (EtOH removed), and it was not dependent on the Parkin/PINK1 mediated mitophagy pathway. Protein expression of key regulators of mitochondrial fission, phospho-Drp1^Ser616^ and S-OPA1, increased during EtOH treatment and recovered to normal levels after removing EtOH. In addition, the critical role of PGC-1α mediated mitochondrial biogenesis in cellular recovery was revealed: inhibition of PGC-1α using SR-18292 after EtOH removal significantly impeded recovery of mitochondrial damage, regeneration of neurites, and cell survival in a concentration-dependent manner. Taken together, our study showed reversibility of mitochondrial morphological and functional damage in stressed neuronal cells and revealed that PGC-1α mediated mitochondrial biogenesis played a critical role in the cellular recovery. This molecular mechanism could be a target for neuroprotection and neurorescue in neurodegenerative diseases.

## Introduction

Mitochondria are key organelles of eukaryotic cells. They are involved in various essential cellular processes, such as ATP production, integration of metabolism, stress sensing, cellular signaling, and survival [1]. In order to maintain bioenergetic levels needed for cellular homeostasis, mitochondrial morphology and function constantly adapt to changing conditions. Excess stress may overwhelm the adaptive capacity leading to severe mitochondrial damage, causing the release of apoptotic signals that promote cell death [2].

Mitochondrial dynamics is essential to maintain mitochondrial integrity and function, which covers a wide range of pathways, involving biogenesis of healthy mitochondria, fusion, fission, and degradation of damaged mitochondria [3]. Several principal proteins responsible for mitochondrial fusion and fission have been identified. Mitochondrial fission is regulated by dynamin-related protein 1 (Drp1), which translocates from the cytosol into the outer mitochondrial membrane to initiate fission. The activity of Drp1 is regulated by the phosphorylation at two key serine residues. Phosphorylation of Ser616 increases Drp1 activity and leads to mitochondrial fission, whereas phosphorylation of Ser637 inhibits fission [4]. Mitochondrial fusion is regulated by mitofusion-1/2 (MFN1/2) in the outer mitochondrial membrane (OMM) and optic atrophy 1 (OPA1) in the inner mitochondrial membrane (IMM) [5]. OPA1 plays an essential role in both fusion and fission of the IMM. OPA1 regulates IMM fusion via its membrane anchored long isoform (L-OPA1). Cleavage of OPA1 by the stress activated metalloprotease OMA1 generates a short isoform (S-OPA1) that facilitates fission of the inner mitochondrial compartment [6]. Mitochondrial dynamics affect not only mitochondrial morphology, but also mitochondrial biogenesis, which is regulated by the peroxisome proliferator-activated receptor γ coactivator-1 (PGC-1) family. PGC-1a is the first identified PGC-1 family member, and is tightly modulated by many transduction effectors such as adenosine monophosphate-activated protein kinase (AMPK), silent information regulator 1 (SIRT1) and mammalian target of rapamycin (mTOR) [7].

Damaged or dysfunctional mitochondria can be degraded in a selective autophagic process termed mitophagy. It has been suggested that mitophagy contributes to maintaining the quality and quantity of the mitochondrial population in cells [8]. The removal of damaged mitochondria proceeds through the PINK1/Parkin pathway that depends on mitochondrial membrane potential depolarization [9]. PINK1 is a mitochondrial serine/threonine kinase imported into the IMM via the preprotein translocase complexes where it is constitutively and rapidly degraded by the matrix processing peptidase (MPP) and presenilin-associated rhomboid like protein (PARL) [10]. Loss of mitochondrial membrane potential disrupts the degradation of PINK1 and reroutes it to the outer membrane of defective mitochondria, where it recruits Parkin, an E3 ubiquitin ligase. On the mitochondrial surface Parkin ubiquitinates a specific subset of OMM proteins, including MFN1 and MFN2, leading to the engulfment of impaired mitochondria by autophagosomes, which fuse with lysosomes causing the eventual elimination of damaged mitochondria [11].

Neuronal survival critically depends on the integrity and functionality of mitochondria [12]. Structural and functional changes, which mitochondria undergo during the cell degeneration process, have been described in detail [13, 14]. Previously, we studied the cell death process in neuronal PC12 cells by exposing them to cell death triggers such as ethanol (5% v/v). Ethanol is a known cell death trigger for in vitro studies by inducing oxidative stress [15]. In addition, ethanol is recognized as a contributing factor to the risk of neurodegenerative diseases [16]. During the cell death process, mitochondria showed many changes and signs of damage such as increased mitochondrial fragmentation, impaired calcium influx, accumulation of reactive oxygen species (ROS), and membrane potential loss. Strikingly, all these mitochondrial injuries could be reversed by timely removing the cell death trigger. This led not only to recovery of the mitochondria but also to the recovery and survival of the neuronal cells. Our data also suggested that this kind of recovery is an intrinsic capacity of cells and does not depend on any exogenously added growth factor [17]. Moreover, this reversible mitochondrial impairment has been observed in diverse neuronal cell lines [18]. Yet, how the recovery of mitochondrial damage relates to the recovery and survival of the cells remains to be elucidated. Identifying the molecular changes occurring in cells undergoing this remarkable recovery from significant mitochondrial damage is a critical step toward possible exploitation of this recovery and survival mechanism for therapeutic benefit. As introduced above, we propose that the signaling pathways that regulate mitochondrial dynamics (fusion and fission, biogenesis and mitophagy) may be involved in the processes of both damage and recovery of mitochondria. Moreover, we propose that these processes are crucial for degeneration and recovery of the neuronal cell itself. In the current study, we further analyzed the morphology and functional damage of mitochondria during cellular degeneration and recovery in the model of ethanol-treated neuronal PC12 cells. We specifically addressed the role of mitochondrial dynamics in both cellular degeneration and recovery, by focusing on gene and protein expression of key regulators of mitochondrial fusion/fission, biogenesis and mitophagy.

## Results

### Reversible loss of neurites induced by EtOH in neuronal PC12 cells

**Fig. 1:**
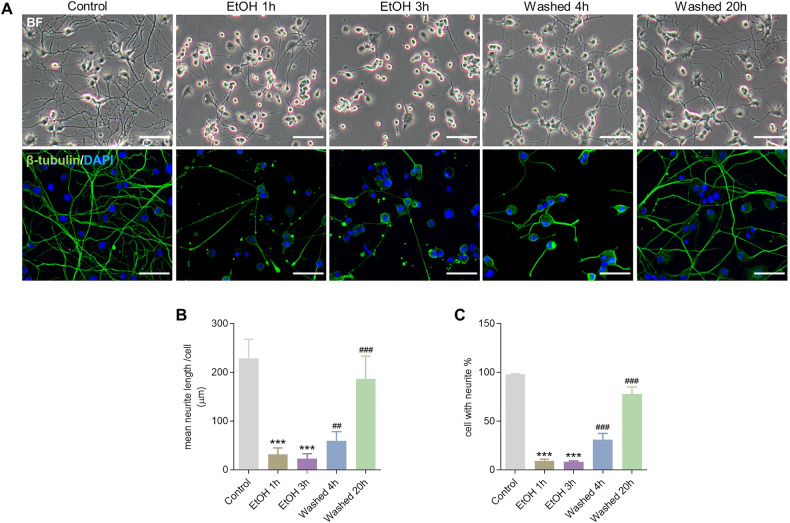
Reversible loss of neurites induced by EtOH in neuronal PC12 cells. **A** Representative bright field (BF) and immunofluorescence images of 5% EtOH treatment (vol/vol, 1 and 3 h) induced neurite retraction, and its regeneration after washing EtOH (Washed, 4 and 20 h) in neuronal PC12 cells. Green: neuron-specific β-III tubulin; Blue: DAPI. Scale bar: 50 µm. **B**, **C** Quantification results of mean neurite length per cell and the percentage of cells with neurites. ^***^P < 0.001, vs. control group; ^##^*P* < 0.01, ^###^*P* < 0.001, vs. EtOH 3 h group. EtOH ethanol.

As shown in Fig. 1A, EtOH (5%, vol/vol) treatment for 1 and 3 h induced significant (EtOH 1 h, *P* < 0.001; EtOH 3 h, *P* < 0.001) neurite loss in neuronal PC12 cells after evaluating bright field images and neuron-specific β-III tubulin staining to mark neurites. Interestingly, by removing EtOH and further culturing cells in fresh culture medium for 4 and 20 h, most of the neuronal PC12 cells that had lost neurites, quickly regrew new neurites with lengths (Washed 4 h, *P* < 0.001; Washed 20 h, *P* < 0.001) comparable to those of control cells (Fig. 1B, C).

### Mitochondrial morphological and functional changes in the process of cellular degeneration and recovery

**Fig. 2:**
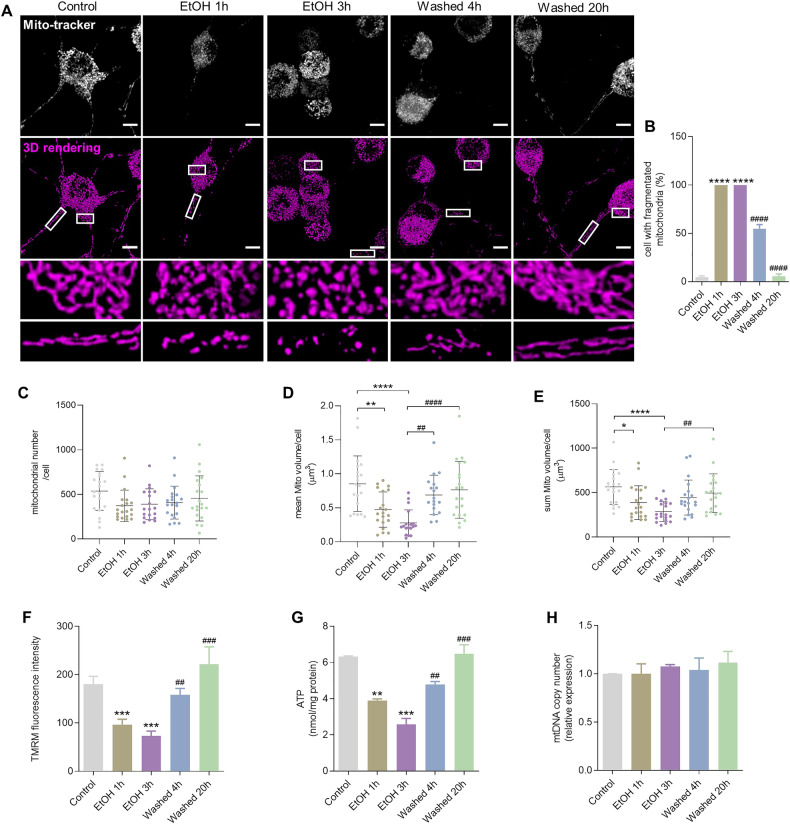
Reversible changes in mitochondrial morphology and function. **A** Original fluorescence and 3D reconstruction images of Mito-tracker stained mitochondria in neuronal PC12 cells showed mitochondrial fragmentation induced by exposure to EtOH (5%, vol/vol, 1 and 3 h) and subsequent recovery of mitochondrial morphology after washing EtOH (Washed, 4 and 20 h). Scale bar: 20 µm. **B** Quantification result of the percentage of cells with mitochondrial fragmentation. Data are presented as mean ± SEM. ^****^P < 0.0001, vs. control group; ^####^*P* < 0.0001, vs. EtOH 3 h group. **C**–**E** Quantification results of mitochondrial number, mean mitochondrial volume and total mitochondrial volume per cell. Data are presented as mean ± SD. ^*^P < 0.05, ^**^P < 0.01, ^****^P < 0.0001, vs. control group; ^##^*P* < 0.01^, ####^*P* < 0.0001, vs. EtOH 3 h group. **F** Quantification of mean fluorescence intensity stained with TMRM. Data are presented as mean ± SEM. ^***^P < 0.001, vs. control group; ^##^*P* < 0.01, ^###^*P* < 0.001, vs. EtOH 3 h group. **G** ATP content (nmol/mg protein) in neuronal PC12 cells. Data are presented as mean ± SEM. ^**^P < 0.01, ^***^P < 0.001, vs. control group; ^##^*P* < 0.01, ^###^*P* < 0.001, vs. EtOH 3 h group. **H** The relative mtDNA copy number estimated with qPCR measurement. Data are presented as mean ± SEM. EtOH ethanol.

Live cell imaging of Mito-tracker staining and representative 3D images showed that mitochondria formed a filamentous network in both soma and neurite in healthy neuronal PC12 cells in control conditions (Fig. 2A). After exposure to EtOH for 1 or 3 h, these large mitochondria fragmented into small, round mitochondria. Notably, after removing EtOH by washing and further culturing cells in fresh culture medium for another 4 or 20 h, cells regained a filamentous mitochondrial morphology, similar to the morphology observed in control cells. During this mitochondrial recovery, neurite regrowth occurred. Quantification showed that, almost 100% of the cells had mitochondrial fragmentation after 1 or 3 h exposure to EtOH (*P* < 0.0001), while more than 90% of the cells regained normal mitochondrial morphology after removing EtOH (*P* < 0.0001) (Fig. 2B).

We quantified the mitochondrial volume and found that the mean volume per mitochondrion significantly decreased during EtOH treatment (EtOH 1 h, *P* < 0.01; EtOH 3 h, *P* < 0.0001), and increased and returned to normal after removing EtOH (Washed 4 h, *P* < 0.01; Washed 20 h, *P* < 0.0001) (Fig. 2D). This is consistent with the recovery of mitochondrial fragmentation that was observed in fluorescence imaging. The total mitochondrial volume of each cell decreased significantly during exposure to EtOH (EtOH 1 h, *P* < 0.05; EtOH 20 h, *P* < 0.0001), which also increased again after removing EtOH (Washed 20 h, *P* < 0.01) (Fig. 2E). Meanwhile, we found that the number of mitochondria per cell decreased slightly but had no significant changes during both mitochondrial fragmentation and recovery (Fig. 2C).

TMRM staining showed that fragmentation of mitochondria was accompanied by loss of mitochondrial membrane potential (EtOH 1 h, *P* < 0.01; EtOH 3 h, *P* < 0.01), which also recovered to normal after removing EtOH (Washed 4 h, *P* < 0.01; Washed 20 h, *P* < 0.001) (Fig. 2F). Intracellular ATP content was then investigated with the ATP assay kit. We found that the levels of intracellular ATP content significantly decreased after exposure to EtOH (EtOH 1 h, *P* < 0.01; EtOH 3 h, *P* < 0.001) and recovered to the normal level after washing EtOH (Washed 4 h, *P* < 0.01; Washed 20 h, *P* < 0.001) (Fig. 2G), in agreement with the TMRM staining results. No significant changes were observed with mitochondrial DNA (mtDNA) copy number during mitochondrial fragmentation and recovery (Fig. 2H).

**Fig. 3:**
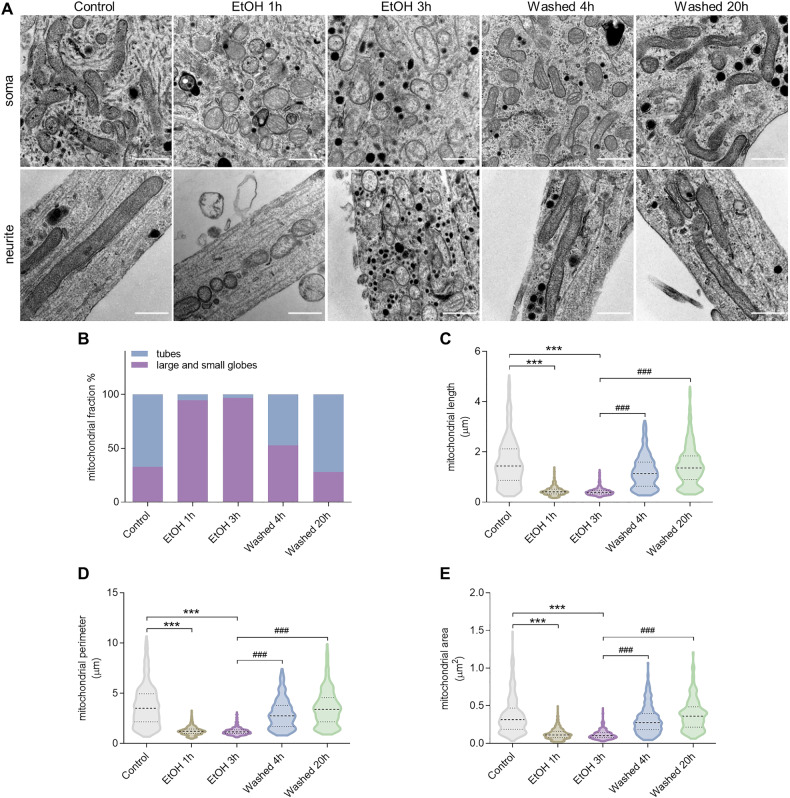
Ultrastructure of mitochondria revealed by transmission electron microscopy in neuronal PC12 cells. **A** Representative images of mitochondrial ultrastructure of neuronal PC12 cells showed EtOH (5%, vol/vol, 1 and 3 h) induced mitochondrial changes such as fragmentation and swelling, and mitochondrial recovery after washing EtOH (Washed, 4 and 20 h). Scale bar: 0.5 µm. **B** The percentage of mitochondria which appeared as tubes or large and small globes in the sections. **C**–**E** Quantification of mitochondria related parameters, including mitochondrial length, perimeter, and area per mitochondrion. At least 300 mitochondria were quantified in each group. Data are presented as mean ± SD. ^***^P < 0.001, vs. control group; ^###^*P* < 0.001, vs. EtOH 3 h group. EtOH ethanol.

In electron microscopy, mitochondria in the control group mainly appeared with a tubular or round profile in cross-sections of both soma and neurite (Fig. 3A). By classifying the mitochondria into tubules and globules as previously reported [19], quantification showed that tubular mitochondria were predominant in the control group (Fig. 3B). In cells treated with EtOH for 1 or 3 h, the mitochondrial structure was significantly damaged; mitochondria appeared as small, round or oval structures with a low matrix density, and reduced or damaged cristae that were broken or swollen. In the cellular degeneration phase, EtOH treatment for 1 or 3 h, the fraction of globular mitochondria increased to 95% and 97%, respectively (Fig. 3B). Notably, in the washed groups, most of the mitochondria regained the elongated tubular morphology. Quantitative analysis showed significant decrease of mitochondrial area (EtOH 1 h, *P* < 0.001; EtOH 3 h, *P* < 0.001), length (EtOH 1 h, *P* < 0.001; EtOH 3 h, *P* < 0.001), and perimeter (EtOH 1 h, *P* < 0.001; EtOH 20 h, *P* < 0.001) in EtOH-treated neuronal PC12 cells compared with control group. In the washed group, mitochondrial area (Washed 4 h, *P* < 0.001; Washed 20 h, *P* < 0.001), length (Washed 4 h, *P* < 0.001; Washed 20 h, *P* < 0.001), and perimeter (Washed 4 h, *P* < 0.001; Washed 20 h, *P* < 0.001) all recovered to levels similar to those in normal cells (Fig. 3C–E).

### Autophagy is activated during cellular degeneration, but not during recovery

**Fig. 4:**
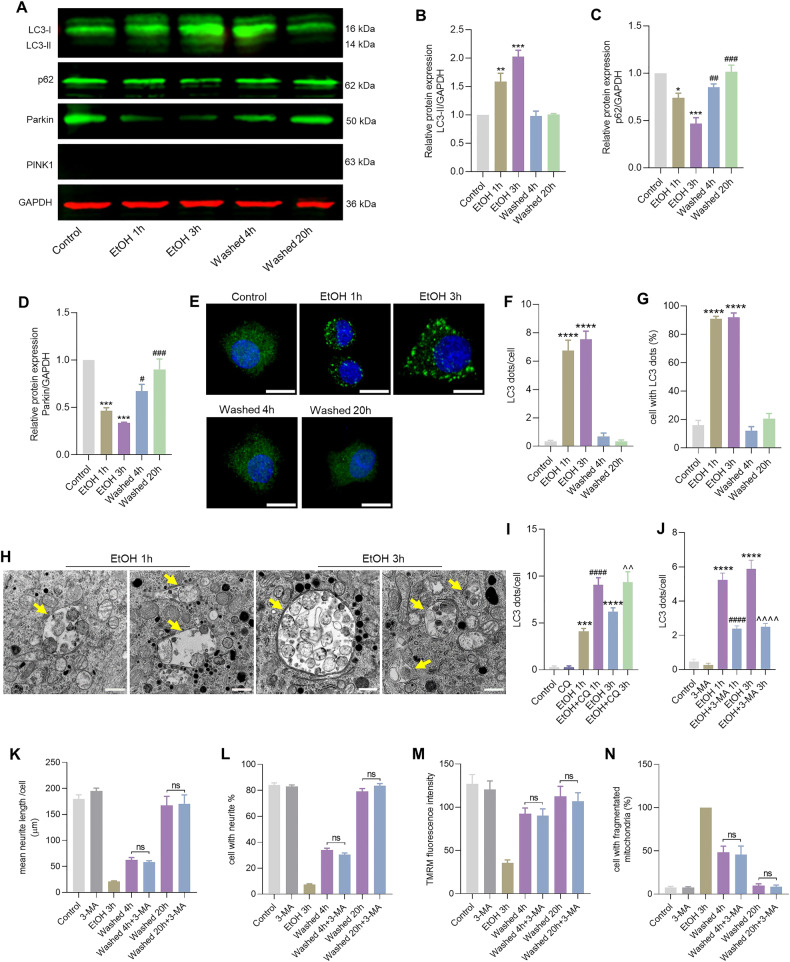
Autophagy is activated during EtOH treatment, but not in the recovery process. **A**–**D** Protein expression of LC3-I/II, p62, Parkin, PINK1 in neuronal PC12 cells detected by western blot. GAPDH was used as a loading control. **E** Immunofluorescence images represent LC3 dots (green) and nuclei (blue) in neuronal PC12 cells. Scale bar: 10 µm. **F**, **G** Quantification of the number of LC3 dots per cell and the percentage of cells with LC3 dots. Data are presented as mean ± SEM. ^****^P < 0.0001, vs. control group. **H** Electron microscopy showed autophagy induced by EtOH (5%, vol/vol, 1 and 3 h) in neuronal PC12 cells. The arrows point to the autophagosome (double membrane) and autolysosome (single membrane). Scale bar: 1 µm. **I**, **J** Quantification of the number of LC3 dots per cell. Data are presented as mean ± SEM. ^***^P < 0.001^, ****^P < 0.0001, vs. control group. ^####^*P* < 0.000, vs. EtOH 1 h group. ^^^^*P* < 0.01, ^^^^^^*P* < 0.0001, vs. EtOH 3 h group. **K**–**N** Quantification results of mean neurite length per cell, the percentage of cell with neurite, the percentage of cells with fragmented mitochondria, and TMRM mean fluorescence intensity, which show that the autophagy inhibitor 3-MA had no inhibitory effect on the cell recovery from EtOH induced damage. EtOH ethanol.

Autophagy often occurs following damage or stress induced mitochondrial fragmentation and mitochondrial membrane potential loss [20]. We investigated whether autophagy was activated by measuring microtubule-associated protein 1 A/1B-light chain 3 (LC3). LC3 has two isoforms, 16-kDa isoform LC3-I and 14-kDa isoform LC3-II. LC3-I is located in the cytosol and after conjugation to phosphatidylethanolamine, converts to the LC3-II isoform, which is present in autophagosome [21]. Western blot results showed that EtOH treatment increased the LC3-I transformation to autophagosome-associated isoform LC3-II (EtOH 1 h, *P* < 0.01; EtOH 3 h, *P* < 0.001). However, in the process of mitochondrial recovery after washing EtOH, no significant LC3-II protein was detected (Fig. 4A, B). Similarly, immunofluorescent localization analysis revealed an increase in the number of LC3 dots per cell (EtOH 1 h, *P* < 0.0001; EtOH 3 h, *P* < 0.0001) and the percentage of cells with LC3 dots during EtOH treatment (EtOH 1 h, *P* < 0.0001; EtOH 3 h, *P* < 0.0001). These parameters returned to normal levels after removing EtOH (Fig. 4E–G). P62 is another marker of autophagy. This protein is lost at the final stage of autophagy during autolysosome degradation [21]. The Western blot showed that there was a decreased protein level of p62 during EtOH treatment (EtOH 1 h, *P* < 0.0001; EtOH 3 h, *P* < 0.0001), which increased to a normal level again during mitochondrial recovery, after removing EtOH (Washed 4 h, *P* < 0.01; Washed 20 h, *P* < 0.001) (Fig. 4A). Transmission electron microscopy further confirmed activation of autophagy in neuronal PC12 cells after EtOH treatment for 1 and 3 h. The arrows point to the autophagosome (double membrane) and autolysosome (single membrane) (Fig. 4H). Next, we investigated whether EtOH induced accumulation of LC3-II and increase in LC3 dots were due to increase in autophagic flux or due to prevention of autophagic proteolysis. For this, we used two drugs: 3-methyladenine (3-MA), a phosphoinositide 3-kinase (PI3K) inhibitor known to inhibit the induction of autophagy, and chloroquine (CQ), a lysosomal inhibitor, to study autophagic flux [22]. Immunofluorescence analysis of the number of LC3 dots showed that 3-MA and CQ effectively inhibited the autophagy response induced by EtOH (Fig. 4I, J). Together these results indicated that autophagy was activated during EtOH treatment when it induced mitochondrial damage, but not in the process of mitochondrial recovery, after washing EtOH. Further study with the autophagy inhibitor 3-MA, showed that 3-MA had no inhibitory effect on both neurite regeneration and the recovery of mitochondrial fragmentation and membrane potential loss (Fig. 4K–N).

The protein expression of Parkin decreased in the degeneration phase (EtOH treatment), which increased again during recovery (EtOH removed) (Fig. 4A, D). No binding of PINK1 was detected during both mitochondrial degeneration and recovery (Fig. 4A). With an alternative PINK1 antibody (PA1-16604, Invitrogen, USA), we also failed to detect PINK1 in this model. These results indicated that EtOH induced autophagy in neuronal PC12 cells in a Parkin/PINK1 independent way.

### Mitochondrial fission and fusion in the process of cellular degeneration and recovery

**Fig. 5:**
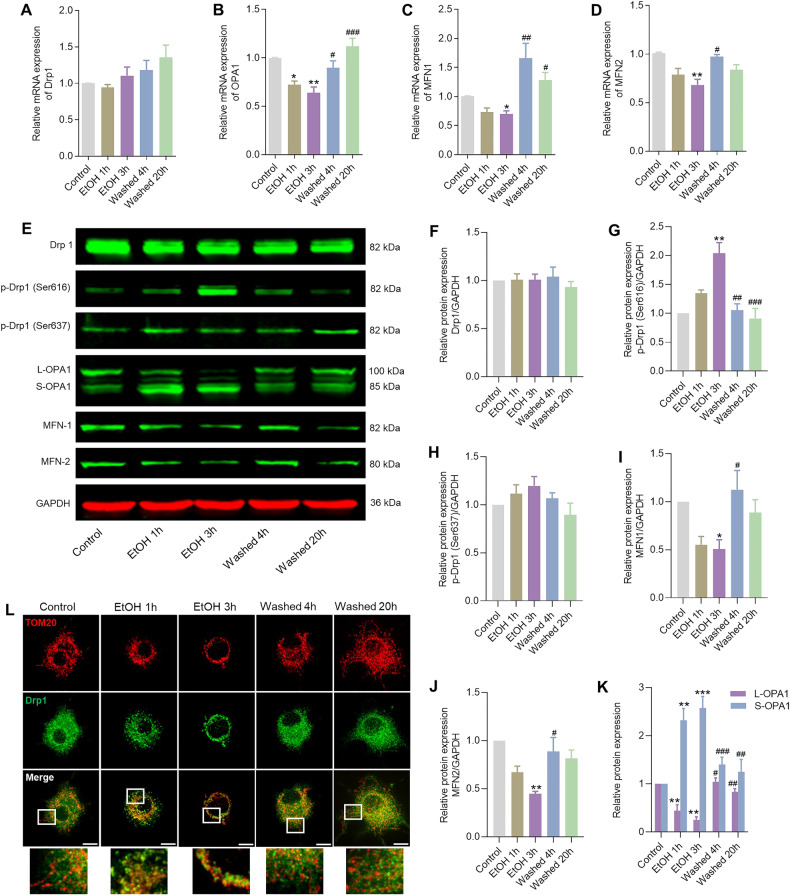
Changes in regulators of mitochondrial fusion/fission in the process of cellular degeneration and recovery. **A**–**D** mRNA expression of Drp1, OPA1, MFN1, and MFN2 measured by qPCR in neuronal PC12 cells. Data are presented as mean ± SEM. ^*^P < 0.05, ^**^P < 0.01^,^ vs. control group; ^#^*P* < 0.05, ^##^*P* < 0.01, ^###^*P* < 0.001, vs. EtOH 3 h group. **E**–**K** Protein expression of Drp1, p-Drp1 (Ser616), p-Drp1 (Ser637), OPA1, MFN1, and MFN2 in neuronal PC12 cells detected by Western blot. Data are presented as mean ± SEM. ^*^P < 0.05, ^**^P < 0.01, ^***^P < 0.001, vs. control group; ^#^*P* < 0.05, ^##^*P* < 0.01^, ###^*P* < 0.001, vs. EtOH 3 h group. **L** Immunofluorescence images showed that Drp1 signals were diffused in the cytosol before EtOH treatment. After applying EtOH for 3 h, the signals became more punctate and appeared to be translocated to the mitochondria. After removing EtOH by washing, the Drp1 signals were again diffused throughout the cytosol. Mitochondria and Drp1 were stained with TOM20 antibody (red) and Drp1 antibody (green) separately. Scale bar: 10 µm. EtOH ethanol.

As shown in Fig. 5, significant changes were observed neither in mRNA nor in protein expression of Drp1 (Fig. 5A, E, F), which is known as the executioner of mitochondrial fission [4]. In contrast to total Drp1 protein, the level of phospho-Drp1^Ser616^ was significantly increased after exposure to EtOH for 3 h, indicating increased Drp1 activity, which decreased to the normal level after washing EtOH for 4 or 20 h (Fig. 5E, G). At the same time, the protein expression of phospho-Drp1^Ser637^, whose phosphorylation inhibits mitochondrial fission, showed no significant changes during EtOH treatment, and after removing EtOH (Fig. 5E, H).

We studied the subcellular localization of Drp1 by immunofluorescence staining, since it has been reported that mitochondrial translocation of Drp1 is a prerequisite for the induction of mitochondrial fission [4]. As shown in Fig. 5L, Drp1 signals were diffused in the cytosol before EtOH treatment. After applying EtOH for 3 h, the signals became more punctate and appeared to be translocated to the mitochondria. After removing EtOH by washing, the Drp1 signals were again diffused throughout the cytosol. This result suggested that mitochondrial translocation of Drp1 is required for EtOH induced mitochondrial fission.

Figure 5B shows that the mRNA expression of OPA1 was significantly decreased after EtOH treatment and increased again after washing EtOH. Western blotting detected two OPA1 isoforms, the L-OPA1 (100 kDa) and S-OPA1 (85 kDa). EtOH treatment for 1 or 3 h resulted in a significant conversion of L-OPA1 to S-OPA1, which also recovered to the levels of the control group when EtOH was removed (Fig. 5E, K). Moreover, the mRNA and protein expression of MFN1 and MFN2, which are required for fusion of the mitochondrial outer membrane, were significantly decreased after EtOH treatment for 3 h, and increased when EtOH was removed, especially at the time point of 4 h after washing EtOH (Fig. 5C–E, I, J).

### Mitochondrial biogenesis promotes mitochondrial recovery, neurite regeneration and cell survival

**Fig. 6:**
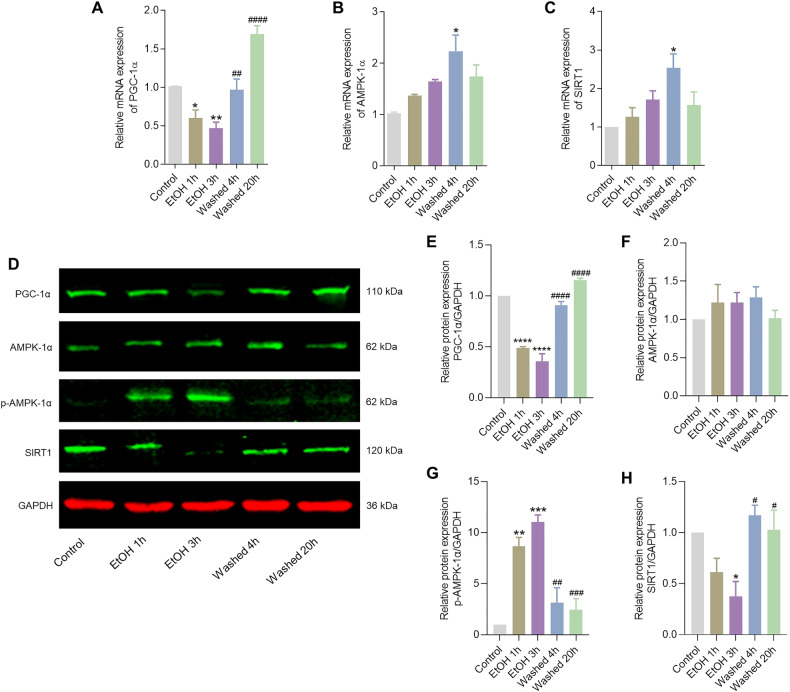
Changes in PGC-1α mediated mitochondrial biogenesis in the process of cellular degeneration and recovery. **A**–**C** mRNA expression of PGC-1α, AMPK-1α, SIRT1 in neuronal PC12 cells measured by qPCR. Data are presented as mean ± SEM. ^*^P < 0.05, ^**^P < 0.01, vs. control group; ^##^*P* < 0.01, ^####^*P* < 0.0001, vs. EtOH 3 h group. **D**–**H** Protein expression of PGC-1α, AMPK-1α, p-AMPK-1α, and SIRT1 in neuronal PC12 cells detected by western blot. Data are presented as mean ± SEM. ^*^P < 0.05, ^**^P < 0.01, ^***^P < 0.001, ^****^P < 0.0001, vs. control grou*p*; ^#^*P* < 0.05, ^##^*P* < 0.01, ^###^*P* < 0.001, ^####^*P* < 0.0001, vs. EtOH 3 h group. EtOH ethanol.

Our measurements showed that there was significant decrease in PGC-1α expression at both the mRNA (EtOH 1 h, *P* < 0.05; EtOH 3 h, *P* < 0.01) and protein (EtOH 1 h, *P* < 0.0001; EtOH 3 h, *P* < 0.0001) level in neuronal PC12 cells upon EtOH treatment. After removing EtOH, PGC-1α mRNA (Washed 4 h, *P* < 0.01; Washed 20 h, *P* < 0.0001) and protein (Washed 4 h, *P* < 0.0001; Washed 20 h, *P* < 0.0001) expression increased markedly during mitochondrial recovery and neurite regeneration (Fig. 6A, D).

We next studied the mRNA and protein expression of AMPK and SIRT1, two metabolic sensors that have been reported to directly regulate the activity of PGC-1α [7]. As shown in Fig. 6B, C, EtOH stimulation did not alter mRNA expression of AMPK and SIRT1; significant increase was only detected when EtOH was removed for 4 h. Western blot analysis revealed that the expression of total AMPK did not change. However, the level of phospho-AMPK significantly increased during exposure to EtOH and decreased to the normal level when EtOH was removed (Fig. 6D, F, G). Moreover, protein expression of SIRT1 was markedly reduced in the EtOH treatment group, and increased again during mitochondrial recovery when EtOH was removed (Fig. 6D, H).

**Fig. 7:**
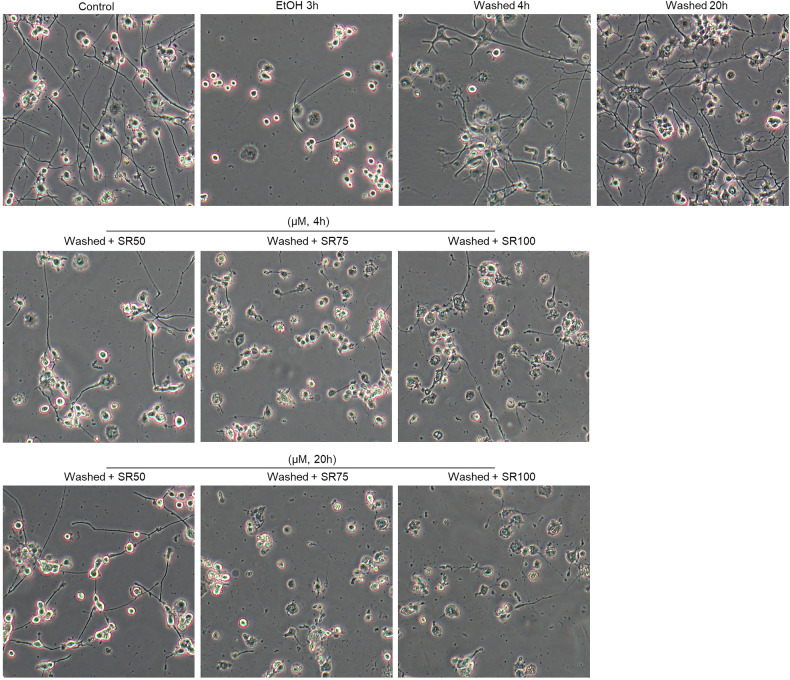
The inhibition effect of SR-18292 on the cell survival and neurite regrowth from EtOH induced damage in neuronal PC12 cells. Bright field images of neuronal PC12 cells with or without EtOH treatment for 3 h, and after washing EtOH then further culturing cells in fresh medium for another 4 or 20 h with or without different concentration of SR-18292 (50, 75, 100 µM). EtOH ethanol.

**Fig. 8:**
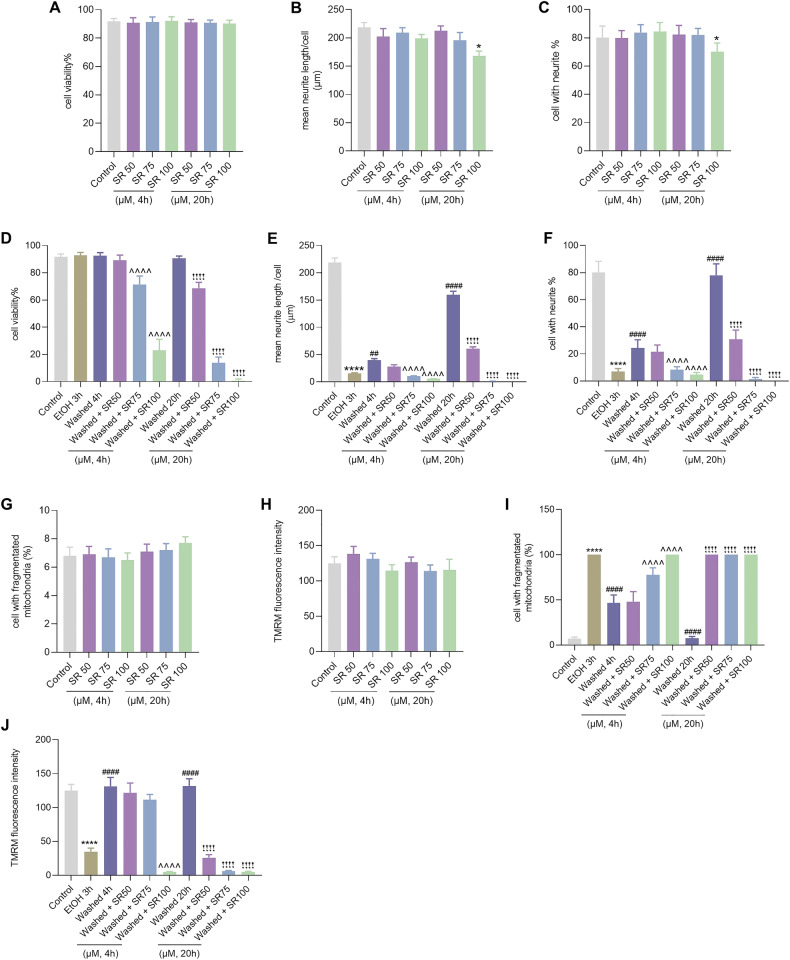
Inhibitory effect of SR-18292 on cell survival and recovery from EtOH induced damage in neuronal PC12 cells. **A**–**C** Quantification of cell viability, the percentage of cells with neurites, and the mean neurite length per cell in neuronal PC12 cells treated alone with different concentration of SR-18292 (50, 75, 100 µM) for 4 and 20 h. Data are presented as mean ± SEM. *P < 0.05, vs. control group. **D**–**F** Quantification of cell viability, the percentage of cells with neurites, and the mean neurite length per cell in neuronal PC12 cells with corresponding treatment. Data are presented as mean ± SEM. ^****^P < 0.0001, vs. control group; ^##^*P* < 0.01, ^####^*P* < 0.0001, vs. EtOH 3 h group; ^^^^^^*P* < 0.0001, vs. Washed 4 h group; ^!!!!^*P* < 0.0001, vs. Washed 20 h group. **G**, **H** Quantification of the percentage of cells with fragmented mitochondria and the TMRM mean fluorescence intensity in neuronal PC12 cells treated alone with different concentrations of SR-18292 (50, 75, 100 µM) for 4 and 20 h. **I**, **J** Quantification of cell viability, the percentage of cells with neurites, and the mean neurite length per cell in neuronal PC12 cells with corresponding treatment. Data are presented as mean ± SEM. ^****^P < 0.0001, vs. control group; ^####^*P* < 0.0001, vs. EtOH 3 h group; ^^^^^^*P* < 0.0001, vs. Washed 4 h group; ^!!!!^*P* < 0.0001, vs. Washed 20 h group.

To clarify whether mitochondrial biogenesis promotes cell recovery and neurite regeneration via PGC-1α activation, we studied the effects of SR-18292, a potent PGC-1α inhibitor [23], on cell recovery from mitochondrial injury and neurite regeneration in neuronal PC12 cells. We first examined the dose effect of SR-18292 on cell viability and neurite maintenance. As shown in Fig. 8A, treatment alone with different concentrations of SR-18292 (50, 75, 100 µM) for 4 or 20 h had no influence on cell viability of neuronal PC12 cells. However, high concentration of SR (100 µM) induced a reduction of the percentage of cells with neurites by 10% (*P* < 0.05) and a decrease of the mean neurite length per cell by 50 µm (*P* < 0.05), indicating that PGC-1α is important for the maintenance of neurites in differentiated neuronal PC12 cells (Fig. 8B, C). Figure 8D showed that EtOH treatment for 3 h had no significant influence on the viability of cells. After removing EtOH and further culturing cells in fresh medium even without NGF and horse serum for another 4 and 20 h, also no further decrease in cell viability was noticed. Strikingly, SR-18292 strongly suppressed the cell survival and neurite regeneration in a dose-dependent manner when cells were further cultured in medium with SR-18292 after washing EtOH (Figs. 7 and 8D–F). At the time point of 4 h after EtOH removal, SR-18292 (75 and 100 µM) incubation had induced cell death by 29.5% (*P* < 0.0001) and 77.1% (*P* < 0.0001), respectively, compared with washed group without SR-18292 at this time point. At the time point of 20 h, SR-18292 (50, 75, and 100 µM) induced cell death by 31.4% (*P* < 0.0001), 86.2% (*P* < 0.0001), and 100% (*P* < 0.0001), respectively, compared with the washed group without SR-18292 at 20 h. Furthermore, the same concentrations of SR-18292 had no influence on mitochondrial morphology and membrane potential in neuronal PC12 cells when applied alone, without EtOH, for 4 and 20 h (Fig. 8G, H); however, they inhibited cell recovery from mitochondrial fragmentation and membrane potential loss dramatically (Fig. 8I, J). These data indicated that PGC-1α mediated mitochondrial biogenesis played an important role during the cell recovery process.

## Discussion

**Fig. 9:**
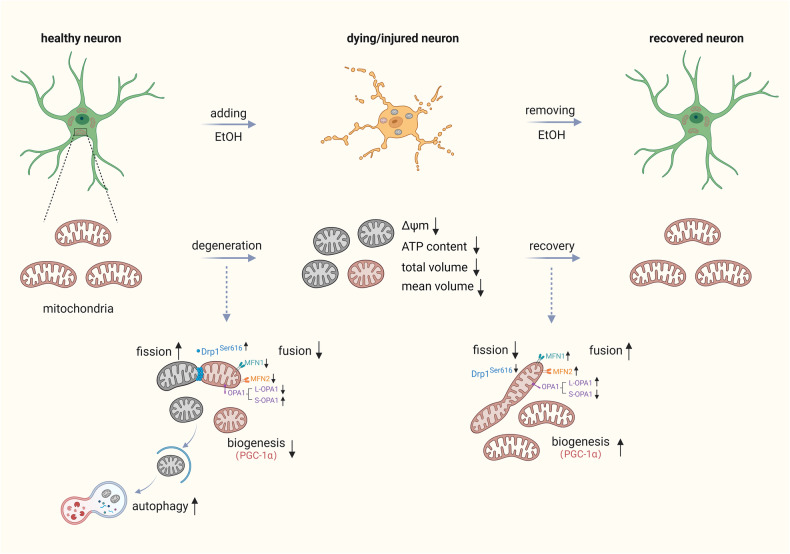
Illustration of mitochondrial dynamics during degeneration and recovery of neuronal PC12 cells. EtOH treatment for 3 h already induced cellular changes and damage such as reactive oxygen species generation, elevation of intracellular Ca^2+^, phosphatidylserine exposure, nuclear shrinkage, DNA damage, mitochondrial fragmentation and mitochondrial membrane potential loss, and retraction of neurites [17]. These phenomena are often associated with regulated cell death. Importantly, after removing ethanol and further culturing the dying cells in fresh culture medium, cells recovered from all these cellular injuries and generated new neurites. In the phase of EtOH induced cellular degeneration, the expression of phospho-Drp1^Ser616^ and S-OPA1 that are related to mitochondrial fission were increased. The expression of MFN1, MFN2, L-OPA1, and PGC-1α that are related to mitochondrial fusion and biogenesis were decreased. Meanwhile, autophagy was activated. In the phase of cellular recovery after removing EtOH, mitochondrial fusion and biogenesis increased and recovered. Inhibiting mitochondrial biogenesis by PGC-1α inhibitor SR-18292 showed remarkable inhibition of not only recovery of mitochondria, but also of neurite regeneration and cell survival. These results indicate that PGC-1α mediated mitochondrial biogenesis may be a potential target for rescuing dying/injured neurons in neurodegenerative diseases.

In the present study, we report reversible morphological and functional damage of mitochondria along with cellular degeneration and recovery in EtOH-treated neuronal PC12 cells. Our mechanistic investigations found the involvement of processes governing mitochondrial dynamics, including mitochondrial fission/fusion, mitophagy, and biogenesis, in both the process of cellular degeneration and its subsequent recovery. Notably, our findings reveal the pivotal role of PGC-1α-mediated mitochondrial biogenesis in the process of cellular recovery from mitochondrial damage, neurite retraction, and overall cell survival (Fig. 9).

A previous study in neuronal PC12 cells showed that, by removing the cell death stimulus EtOH, dying cells could survive and recover from diverse apoptotic events that are related to neuronal cell death, including neurite retraction, nuclear condensation, DNA damage, mitochondrial fragmentation, mitochondrial membrane potential loss, increased ROS generation and increased level of intracellular Ca^2+^ [17]. In the present study, we analysed the mitochondrial ultrastructure in EtOH-treated neuronal PC12 cells and found fragmented mitochondria with swollen structure, disrupted cristae, and less electron-dense mitochondrial matrix (Fig. 3), like the damage of mitochondria described in neurodegenerative disease patients [24–26]. Importantly, these damaged mitochondria regained their tubular network with normal cristae and matrix density when the cell death stimulus EtOH was removed (Fig. 3). The fragmentation of mitochondria not only occurred together with significant membrane potential loss, but also with decreased ATP production and total mitochondrial volume (Fig. 2). These results are consistent with previous studies that mitochondrial dynamics, as well as ultrastructure and volume, are tightly linked to mitochondrial function [27]. Notably, all these mitochondrial parameters recovered to the normal levels of the control group after removing EtOH. Meanwhile, the mtDNA copy number showed no significant changes neither during EtOH treatment nor during cell recovery after washing EtOH (Fig. 2H). This result is puzzling since mtDNA number has been proposed as a biomarker of mitochondrial function and decreased mtDNA number was observed in a variety of neurodegenerative disorders [28–32]. Our data may suggest that while morphology and function of mitochondria undergo massive changes, most of the mtDNA is retained and may contribute to cellular recovery.

Mitophagy, a type of selective autophagy, is an important mitochondrial dynamics mechanism which eliminates damaged mitochondria, and often becomes active following damage or stress induced mitochondrial fragmentation and membrane potential loss [20]. The Parkin/PINK1 mediated mitophagy has been shown to have a protective effect for patients with neurodegenerative disease [33]. Here, we hypothesized that mitophagy is activated to remove the damaged mitochondria and provide energy to promote the cell recovery from mitochondrial defects. However, activated mitophagy was observed exclusively during the phase of cellular degeneration, no detectable autophagic activity was evident in the process of cellular and mitochondrial recovery following the removal of EtOH (Fig. 4). Moreover, the use of an autophagy inhibitor demonstrated no inhibitory effect on the recovery of mitochondrial damage and neurite regrowth (Fig. 4K–N). These results imply that autophagy was activated specifically to eliminate damaged mitochondria during EtOH treatment. However, it does not appear to play a pivotal role during the subsequent process of cellular and mitochondrial recovery. In addition, the protein expression of Parkin exhibited a decrease during EtOH treatment, and no band corresponding to PINK1 was detected. Notably, PINK1 is constitutively degraded by PARL in the mitochondria, resulting in undetectable basal levels of PINK1 [10]. These data are not consistent with previous studies, in which there was a higher level of protein expression of Parkin and PINK when mitophagy was activated [34–36]. The findings suggest that the observed mitophagy was not mediated via the Parkin/PINK1 pathway. Parkin, known as a multifunctional ubiquitin E3 ligase, participates in various cellular processes. In addition to its role in regulating mitophagy, studies have demonstrated that Parkin can enhance mitochondrial biogenesis, preserve mitochondrial genome integrity, and regulate mitochondrial dynamics [37–39]. Therefore, the decreased expression of Parkin observed after EtOH treatment in neuronal PC12 cells and its subsequent increase upon EtOH removal may rather point at involvement of Parkin in the recovery process by these other roles of Parkin.

Quantitative analysis of the electron microscopy revealed that 97% of mitochondria after a 3 h EtOH treatment had globular profiles characterized by damaged cristae and lower matrix density (Fig. 3B). Notably, as autophagy was only detected during EtOH treatment and not in the subsequent phase of mitochondrial and cellular recovery after EtOH removal, we hypothesize that not all fragmented mitochondria were eliminated by autophagy. Extended (up to 15 h) exposure of neuronal PC12 cells to EtOH also demonstrated a further increase in transformation of LC3-I to LC3-II (Supplemental Fig. 1), indicating continuation of autophagy activity and degradation of damaged mitochondria. Based on these findings, it is plausible to infer that at least a portion of the fragmented mitochondria, present after 3 h of EtOH treatment, could undergo re-fusion, leading to the restoration of a normal tubular structure and cristae during the recovery phase. Consistent with these observations, we identified an elevation in the protein expression of phospho-Drp1^Ser616^ and observed the translocation of Drp1 from the cytoplasm to mitochondria following EtOH treatment, providing an explanation for the observed widespread mitochondrial fragmentation (Fig. 5A, L). Meanwhile, there was a notable conversion of L-OPA1 to S-OPA1 returning to normal levels upon EtOH removal (Fig. 5A). L-OPA1, adhering to the inner mitochondrial membrane (IMM), plays a crucial role in IMM fusion, while its proteolytic processing releases S-OPA1 into the intermembrane space, where it facilitates fission of the inner mitochondrial compartment [6]. OPA1 is also implicated in the organization of cristae structure [40]. The restoration of L-OPA1 expression after EtOH removal indicates its involvement in the recovery of mitochondrial network morphology and cristae morphogenesis. Furthermore, cristae structure has been demonstrated to reflect mitochondrial oxidative phosphorylation function, suggesting that the L-OPA1-induced recovery of mitochondrial ultrastructure might be associated with the bioenergetic status, as evidenced by the restored ATP production and mitochondrial membrane potential (Fig. 2F, G).

The decrease in total mitochondrial volume during EtOH treatment and its subsequent recovery (Fig. 2E) suggests the involvement of mitochondrial autophagy and biogenesis. PGC-1α, is recognized as a master regulator of mitochondrial biogenesis [7]. Our results demonstrated the activation of autophagy during EtOH treatment alongside a significant reduction in PGC-1α expression at both the mRNA and protein levels (Fig. 6A, D). Importantly, a substantial increase in PGC-1α expression was noted during the mitochondrial recovery phase after EtOH removal. Moreover, the PGC-1α inhibitor SR-18292 exhibited a remarkable inhibitory effect on mitochondrial recovery, neurite regrowth, and cell viability in a concentration-dependent manner (Figs. 7 and 8). Incubating neuronal PC12 cells with SR-18292 at a concentration of 100 µM after EtOH removal for 20 h resulted in 100% cell death. This is in stark contrast with our previous findings on cell viability that more than 90% neuronal PC12 cells survived and recovered from 3 h exposure to EtOH [17]. It indicates the critical role of PGC-1α-mediated mitochondrial biogenesis in the recovery of neuronal PC12 cells from EtOH-induced cellular degeneration.

Metabolic sensors, including AMPK and SIRT1, have been established as key regulators controlling the activity of PGC-1α [7]. Our findings indicate that AMPK was activated specifically during cellular and mitochondrial degeneration after EtOH treatment but not in the subsequent recovery phase after EtOH removal (Fig. 6D). AMPK serves as a crucial energy sensor, playing a role in maintaining cellular energy homeostasis and becoming activated in response to stresses that deplete cellular ATP supplies [41]. Given its association with autophagy triggered by various cellular stresses, including nutrient deficit [42], the observed AMPK activation in our study aligns with the induction of autophagy but not with increase in PGC-1α, suggesting that in EtOH-treated neuronal PC12 cells, AMPK primarily regulates autophagy rather than mitochondrial biogenesis. Meanwhile, our results showed that the protein expression of SIRT1 follows a pattern parallel to that of PGC-1α (Fig. 6), suggesting that the regulation of PGC-1α may be mediated through SIRT1 activation. However, the precise mechanisms by which SIRT1 regulates PGC-1α activation and promotes cell recovery from mitochondrial abnormalities and cell survival needs further research.

Mitochondria have been implicated in the pathogenesis of neurodegenerative diseases such as Parkinson’s disease (PD), Alzheimer’s disease (AD), Amyotrophic lateral sclerosis (ALS), Huntington’s disease (HD) and glaucoma, where their morphological and functional impairments are associated with disease progression [43–46]. Our results agree well with these findings and extend them by showing that mitochondria are not only important in the process of neuronal cell death but also in the recovery of injured neuronal cells. Particularly mitochondrial biogenesis is important for recovery and survival of injured neurons. This suggests that restoration of proper function and high activity levels of mitochondria may be an important driver of the recovery of degenerating neuronal cells. The importance of mitochondrial biogenesis is underscored by a recent study on human stem cell derived retinal ganglion cells. The findings indicated that mitochondrial biogenesis was important for the recovery of mitochondrial homeostasis and pro-survival responses [47].

Reversible mitochondrial fragmentation was also observed in an in vivo model, specifically in the neocortex of anesthetized mice after ischemic and traumatic injury, as revealed by quantitative two-photon imaging [19]. It is important to note that these findings as well as our in vitro findings were obtained in acute injury models, while neurodegenerative diseases usually involve chronic stress and slow progression of cell death. The question of whether the same mitochondrial phenomena occur in chronic neurodegenerative models requires further dedicated studies.

In conclusion, our study shows that mitochondria play a role both in the degeneration when neuronal cells are exposed to stress and in the recovery when the stress is lifted. One of these reversible changes is the fragmentation of mitochondria and the loss of the mitochondrial membrane potential. This phenomenon is observed in various neuronal cell lines subjected to diverse cell death triggers, indicating that this may be a general feature of neurons. The cell autonomous capacity of recovery via mechanisms of mitochondrial maintenance and quality control may open up new possibilities for therapeutic interventions for neurodegenerative diseases. More specifically, our findings reveal the pivotal role of PGC-1α-mediated mitochondrial biogenesis in the recovery of compromised neurons from mitochondrial damage. Stimulating this pathway or maybe more generally, boosting mitochondrial activity, may represent a potential strategy for rescuing dying or injured neurons in neurodegenerative disorders.

## Materials and methods

### Cell culture

Pheochromocytoma line 12 (PC12) cells were obtained from Leibniz Institute DSMZ (ACC 159) and cultured as described before, with minor modifications [48]. Briefly, PC12 cells were maintained undifferentiated in 85% RPMI 1640 medium (Gibco™ 61870-010, Thermo Fisher, USA) supplemented with 10% horse serum (HS; H1270, Sigma, USA), 5% heat-inactivated fetal bovine serum (FBS; F7524, Sigma, USA), and 1% penicillin-streptomycin (pen-strep) antibiotic (Gibco™ 15140122, Thermo Fisher, USA) at 37 °C in a 5% CO_2_ incubator. To induce differentiation, cells were dissociated and plated on PDL (50 μg/ml; P6407, Sigma, USA) and laminin (10 μg/ml; 3400-010-02, R&D Systems, USA) coated cell culture plate in low serum conditions (1% horse serum) and 50 ng/ml of nerve growth factor (NGF; N1408, Sigma, USA). Cells were differentiated for 6–7 days before using for experiments. Mature neuronal PC12 cells were defined as cells harboring neurites at least two cell body diameters in length [49]. Regular mycoplasma testing was conducted every month to prevent contamination.

### Cell viability measurement

Cell death was determined by Hoechst 33342 (Sigma, USA) and propidium iodide (PI, Sigma, USA) double fluorescent staining as previously described [50]. After treatment, the cells in each group were stained with Hoechst 33342 (5 µg/mL) and PI (5 µg/mL) for 15 min in the dark. The stained cells were observed using Olympus IX81 inverted fluorescence microscopy. To assess cell viability, 20 visual fields were randomly selected from each group and quantified with Fiji.

### Mitochondrial morphology imaging and volume quantification

Mitochondrial structure was visualized by Mito-tracker (M22426, Invitrogen™, USA) staining. After treatment, cells were incubated with Mito-tracker (20 nM) at 37 °C in dark for 30 min, then washed with PBS for three times and covered with fresh medium. Cells were imaged by FEI Corrsight microscope with laser line of 640 nm and the 446/523/600/677 nm BrightLine® quad-band bandpass filter. Mitochondrial volume quantification was done with a similar MATLAB script as previously reported [51] with the modification that our analysis was performed in 3D using the DIPimage toolbox (https://diplib.org/). To quantify the mitochondrial volume, neuronal PC12 cells were imaged with z-stacks consisting of 100 planes and an interval of 0.147 μm. Images were shown in the merged z-stacks of 100 planes with the maximum fluorescence intensity. Representative images were edited in Fiji, while 3D rendering was obtained with the 3D Viewer plugin.

### Mitochondrial membrane potential measurement

Mitochondrial morphology and membrane potential changes were detected by staining cells with tetramethylrhodamine (TMRM, No. 134361, Invitrogen™, USA). After treatment, cells were incubated with TMRM (100 nM) at 37 °C in dark for 30 min, then washed with PBS for three times and covered with fresh medium. Images were acquired by FEI Corrsight microscope with the laser line of 561 nm and a Semrock 446/523/600/677 nm BrightLine® quad-band bandpass filter. Up to 35 fields of view each group were captured. TMRM fluorescence intensity was quantified with Fiji.

### ATP content measurement

ATP content was determined using ATP Assay Kit (ab83355, Abcam, UK) according to manufacturer’s instruction. Briefly, 1.5 × 10^6^ PC12 cells from each group were seeded in a T25 flask and differentiated for 6 days. After treatment, cells were collected by rubber scraping, centrifuged, and incubated with lysis buffer. The luminescence was measured fluorometrically (Ex/Em = 535/587 nm) using CLARIOstar plate reader (BMG LABTECH). ATP content was calculated from a freshly prepared standard curve and normalized to protein content. The protein content was analyzed by the BCA Protein Assay Kit (23225, Thermo Scientific^TM^, USA).

### Electron microscopy

Cells were fixed with 2.5% Glutaraldehyde in 0.1 M phosphate buffer and kept in the fixative for 24 h at 4 °C. Then cells were washed with 0.1 M cacodylate buffer and postfixed with 1% osmium tetroxide in the same buffer containing 1.5% potassium ferricyanide for 1 h in the dark at 4 °C. Then cells were dehydrated in ethanol, infiltrated with Epon resin for 2 days, embedded in the same resin and polymerized at 60 °C for 48 h. Ultrathin sections of 70 nm in thickness were cut using a Leica Ultracut UCT ultramicrotome (Leica Microsystems Vienna) and mounted on Formvar-coated copper grids. Cells were stained with 2% uranyl acetate in water and lead citrate. Then, sections were observed in a Tecnai T12 Electron Microscope equipped with an Eagle 4kx4k CCD camera (Thermo Fisher Scientific). Quantification of mitochondrial-related parameters from transmission electron microscopy (TEM) cross-sections, including mitochondrial length, perimeter, and area were done as previously reported [52] with Fiji.

### Western blot

The proteins were extracted from cells using RIPA lysis buffer (89900, Thermo Scientific™, USA) containing Halt™ Protease and Phosphatase Inhibitor Cocktail (78441, Thermo Scientific™, USA). Proteins were separated by 8%-13% sodium dodecylsulfate polyacrylamide gel electrophoresis (SDS-PAGE). Then proteins were transferred onto nitrocellulose membranes in Trans-Blot Turbo Transfer System (1704150, Bio-Rad, USA). The membrane was blocked with SuperBlock™ Blocking Buffer (37535, Thermo Scientific™, USA) for 30 min at room temperature. After blocking, membranes were incubated overnight at 4°C with primary antibodies against LC3B (NB100-2220, Novus Biologicals, USA), p62/SQSTM1 (NBP1-48320, Novus Biologicals, USA), PINK1 (BC100-494, Novus Biologicals, USA), Parkin (sc-32282, Santa Cruz, USA), Drp1 (8570 S, Cell Signaling Technology, USA), p-Drp1 (Ser616) (PA5-106169, Invitrogen^TM^, USA), p-Drp1 (Ser637) (PA5-101038, Invitrogen^TM^, USA), OPA1 (67589S, Cell Signaling Technology, USA), Mitofusion-1 (14739S, Cell Signaling Technology, USA), Mitofusion-2 (9482S, Cell Signaling Technology, USA), PGC-1α (NB100-60955, Novus Biologicals, USA), AMPK-1α (2532S, Cell Signaling Technology, USA), p-AMPK-1α (2531S, Cell Signaling Technology, USA), SIRT1 (9475S, Cell Signaling Technology, USA), and GAPDH (10R-G109a, Fitzgerald Industries, UK). The secondary antibodies used were IRDye 800CW donkey anti-rabbit IgG (H + L), IRDye 800CW donkey anti-goat, and IRDye 680LT donkey anti-mouse IgG (H + L) (LI-COR Biosciences, USA). The membranes were scanned and analyzed using Odyssey imaging system (LI-COR Biosciences, USA). To quantify the western blotting data, the target proteins in each experiment were firstly normalized to their respective GAPDH intensities, and then the normalized intensities were compared with the intensity of the control group and expressed as relative values to their controls.

### RNA isolation and quantitative reverse transcriptase PCR

The total RNA was prepared from the differentiated PC12 cells using Trizol reagent (15596026, Invitrogen^TM^, USA). The RNA integrity and concentration were measured using a NanoDrop ND-1000 Spectrophotometer. All RNA samples with an OD260/OD280 between 1.8 and 2.0 were used for quantitative real-time PCR. Total RNA was reverse-transcribed with the iScript cDNA Synthesis Kit (1708890, Bio-Rad, USA). Quantitative real-time PCR was performed in the LightCycler ® 480 System (Roche) using the SensiMix™ SYBR® No-ROX (QT650-20, Meridian Bioscience, USA) according to the instructions provided. The run PCR program was as follows: 1 cycle at 95 °C for 10 min, 40 cycles at 95 °C for 15 s, 60 °C for 15 s, and 72 °C for 15 s. Relative gene expression levels were calculated according to the 2^−∆∆CT^ method. The mRNA levels were quantified using GAPDH as a housekeeping gene for normalization. Primer sequences are listed in Supplemental Table 1.

### Mitochondrial DNA copy number measurement

The amount of mitochondrial DNA was determined by quantitative real-time PCR as described previously [53]. Total DNA from cultured cells was extracted with Genomic DNA Extraction Kit (ab156900, Abcam, UK) according to the manufacturer’s instruction. DNA concentration was measured using a NanoDrop ND-1000 Spectrophotometer. PCR was performed with DNA template (10 ng) in 20 μl reaction mixture containing 10 μl SensiMix (QT650-20, Meridian Bioscience) and 2 μl of each primer. The following primers were used: MT-ND1 forward primer: ATTCTAGCCACATCAAGTCTTT, MT-ND1 reverse primer: GGAGGACGGATAAGAGGATAAT, β-actin forward primer: GAAATCGTGCGTGACATTAAAG, β-actin reverse primer: ATCGGAACCGCTCATTG. Relative mitochondrial DNA copy number were calculated according to the 2^−∆∆CT^ method and expressed as mean mtDNA copy number relative to β-actin.

### Immunofluorescence

Cells were fixed in 4% paraformaldehyde (PFA) for 30 min at 4 °C followed by permeabilization in 0.1% Triton X-100 for 10 min at room temperature and blocked with 4% bovine serum albumin (BSA) for 1 h at room temperature. For immunostaining, cells were incubated with primary antibodies against neuron-specific β-III tubulin (MAB1195, R&D systems, USA), LC3B (NB100-2220, Novus Biologicals, USA), TOM20 (42406S, Cell Signaling Technology), and Drp1 (8570S, Cell Signaling Technology) overnight at 4 °C. After carefully rinsing in PBS, cells were incubated with a second antibody conjugated with Alexa 488 or 594 (Invitrogen) for 1 h at room temperature. The nucleus was labeled by DAPI (D9542, Sigma, USA). The cells were then observed and photographed with FEI CorrSight.

### Statistics

Data were obtained from at least three independent experiments and presented as mean ± SD or SEM. Statistical analyses were performed throughout using GraphPad Prism version 9.4.1. For comparison between more than two groups, values were evaluated by one-way ANOVA followed by a Tukey Kramer test. *P* < 0.05 was considered statistically significant.

### Supplementary information

Original full length western blots

Supplementary information

## Data Availability

All data generated during this study are included in this article (and its supplementary information files).

## References

[1] Nunnari J, Suomalainen A (2012). Mitochondria: in sickness and in health. Cell.

[2] Sokolova I (2018). Mitochondrial adaptations to variable environments and their role in animals’ stress tolerance. Integr Comp Biol.

[3] Quintana-Cabrera R, Scorrano L (2023). Determinants and outcomes of mitochondrial dynamics. Mol Cell.

[4] Kraus F, Roy K, Pucadyil TJ, Ryan MT (2021). Function and regulation of the divisome for mitochondrial fission. Nature.

[5] Pernas L, Scorrano L (2016). Mito-morphosis: mitochondrial fusion, fission, and cristae remodeling as key mediators of cellular function. Annu Rev Physiol.

[6] MacVicar T, Langer T (2016). OPA1 processing in cell death and disease - the long and short of it. J Cell Sci.

[7] Canto C, Auwerx J (2009). PGC-1alpha, SIRT1 and AMPK, an energy sensing network that controls energy expenditure. Curr Opin Lipidol.

[8] Lou GF, Palikaras K, Lautrup S, Scheibye-Knudsen M, Tavernarakis N, Fang EF (2020). Mitophagy and Neuroprotection. Trends Mol Med.

[9] Ashrafi G, Schwarz TL (2013). The pathways of mitophagy for quality control and clearance of mitochondria. Cell Death Differ.

[10] Yamano K, Youle RJ (2013). PINK1 is degraded through the N-end rule pathway. Autophagy.

[11] Nguyen TN, Padman BS, Lazarou M (2016). Deciphering the molecular signals of PINK1/Parkin mitophagy. Trends Cell Biol.

[12] Reddy PH, Reddy TP (2011). Mitochondria as a therapeutic target for aging and neurodegenerative diseases. Curr Alzheimer Res.

[13] Guo C, Sun L, Chen X, Zhang D (2013). Oxidative stress, mitochondrial damage and neurodegenerative diseases. Neural Regen Res.

[14] Cenini G, Lloret A, Cascella R (2020). Oxidative stress and mitochondrial damage in neurodegenerative diseases: from molecular mechanisms to targeted therapies. Oxid Med Cell Longev.

[15] Comporti M, Signorini C, Leoncini S, Gardi C, Ciccoli L, Giardini A (2010). Ethanol-induced oxidative stress: basic knowledge. Genes Nutr.

[16] Mooney, SM, Miller, MW, Henderson GI. Intracellular events in ethanol-induced neuronal death. In: Miller MW, editor. Brain development: normal processes and the effects of alcohol and nicotine. England: Oxford University Press; 2006. p. 267–78.

[17] You W, Berendschot T, Knoops K, van Zandvoort M, Webers CAB, Reutelingsperger CPM (2022). Single cell analysis of reversibility of the cell death program in ethanol-treated neuronal PC12 cells. Int J Mol Sci.

[18] You W, Zhou T, Knoops K, Berendschot T, van Zandvoort M, Germeraad WTV (2023). Stressed neuronal cells can recover from profound membrane blebbing, nuclear condensation and mitochondrial fragmentation, but not from cytochrome c release. Sci Rep.

[19] Kislin M, Sword J, Fomitcheva IV, Croom D, Pryazhnikov E, Lihavainen E (2017). Reversible disruption of neuronal mitochondria by ischemic and traumatic injury revealed by quantitative two-photon imaging in the neocortex of anesthetized mice. J Neurosci.

[20] Twig G, Shirihai OS (2011). The interplay between mitochondrial dynamics and mitophagy. Antioxid Redox Sign.

[21] Mizushima N, Yoshimori T, Levine B (2010). Methods in mammalian autophagy research. Cell.

[22] Wu YT, Tan HL, Shui G, Bauvy C, Huang Q, Wenk MR (2010). Dual role of 3-methyladenine in modulation of autophagy via different temporal patterns of inhibition on class I and III phosphoinositide 3-kinase. J Biol Chem.

[23] Sharabi K, Lin H, Tavares CDJ, Dominy JE, Camporez JP, Perry RJ (2017). Selective chemical inhibition of PGC-1alpha gluconeogenic activity ameliorates type 2 diabetes. Cell.

[24] Napoli L, Crugnola V, Lamperti C, Silani V, Di Mauro S, Bresolin N (2011). Ultrastructural mitochondrial abnormalities in patients with sporadic amyotrophic lateral sclerosis. Arch Neurol-Chicago.

[25] Tribble JR, Vasalauskaite A, Redmond T, Young RD, Hassan S, Fautsch MP (2019). Midget retinal ganglion cell dendritic and mitochondrial degeneration is an early feature of human glaucoma. Brain Commun.

[26] Sasaki S (2010). Determination of altered mitochondria ultrastructure by electron microscopy. Methods Mol Biol.

[27] Iovine JC, Claypool SM, Alder NN (2021). Mitochondrial compartmentalization: emerging themes in structure and function. Trends Biochem Sci.

[28] Castellani CA, Longchamps RJ, Sun J, Guallar E, Arking DE (2020). Thinking outside the nucleus: mitochondrial DNA copy number in health and disease. Mitochondrion.

[29] Xu YY, Xu LL, Han M, Liu XT, Li F, Zhou XY (2019). Altered mitochondrial DNA methylation and mitochondrial DNA copy number in an APP/PS1 transgenic mouse model of Alzheimer disease. Biochem Bioph Res Co.

[30] Petersen MH, Budtz-Jorgensen E, Sorensen SA, Nielsen JE, Hjermind LE, Vinther-Jensen T (2014). Reduction in mitochondrial DNA copy number in peripheral leukocytes after onset of Huntington’s disease. Mitochondrion.

[31] Alvarez-Mora MI, Podlesniy P, Riazuelo T, Molina-Porcel L, Gelpi E, Rodriguez-Revenga L (2022). Reduced mtDNA copy number in the prefrontal cortex of C9ORF72 patients. Mol Neurobiol.

[32] Xu Y, Cheng L, Sun J, Li F, Liu X, Wei Y (2021). Hypermethylation of mitochondrial cytochrome b and cytochrome c oxidase II genes with decreased mitochondrial DNA copy numbers in the APP/PS1 transgenic mouse model of Alzheimer’s disease. Neurochem Res.

[33] Du F, Yu Q, Yan SJ, Hu G, Lue LF, Walker DG (2017). PINK1 signalling rescues amyloid pathology and mitochondrial dysfunction in Alzheimer’s disease. Brain.

[34] Samuvel DJ, Li L, Krishnasamy Y, Gooz M, Takemoto K, Woster PM (2022). Mitochondrial depolarization after acute ethanol treatment drives mitophagy in living mice. Autophagy.

[35] Choi GE, Lee HJ, Chae CW, Cho JH, Jung YH, Kim JS (2021). BNIP3L/NIX-mediated mitophagy protects against glucocorticoid-induced synapse defects. Nat Commun.

[36] Bordi M, Darji S, Sato Y, Mellen M, Berg MJ, Kumar A (2019). mTOR hyperactivation in Down Syndrome underlies deficits in autophagy induction, autophagosome formation, and mitophagy. Cell Death Dis.

[37] Shin JH, Ko HS, Kang H, Lee Y, Lee YI, Pletinkova O (2011). PARIS (ZNF746) repression of PGC-1alpha contributes to neurodegeneration in Parkinson’s disease. Cell.

[38] Park J, Lee SB, Lee S, Kim Y, Song S, Kim S (2006). Mitochondrial dysfunction in Drosophila PINK1 mutants is complemented by parkin. Nature.

[39] Rothfuss O, Fischer H, Hasegawa T, Maisel M, Leitner P, Miesel F (2009). Parkin protects mitochondrial genome integrity and supports mitochondrial DNA repair. Hum Mol Genet.

[40] Del Dotto V, Mishra P, Vidoni S, Fogazza M, Maresca A, Caporali L (2017). OPA1 isoforms in the hierarchical organization of mitochondrial functions. Cell Rep.

[41] Mihaylova MM, Shaw RJ (2011). The AMPK signalling pathway coordinates cell growth, autophagy and metabolism. Nat Cell Biol.

[42] Wang S, Li H, Yuan M, Fan H, Cai Z (2022). Role of AMPK in autophagy. Front Physiol.

[43] Olagunju AS, Ahammad F, Alagbe AA, Otenaike TA, Teibo JO, Mohammad F (2023). Mitochondrial dysfunction: a notable contributor to the progression of Alzheimer’s and Parkinson’s disease. Heliyon.

[44] Osborne NN, Nunez-Alvarez C, Joglar B, Del Olmo-Aguado S (2016). Glaucoma: focus on mitochondria in relation to pathogenesis and neuroprotection. Eur J Pharmacol.

[45] Golpich M, Amini E, Mohamed Z, Azman Ali R, Mohamed Ibrahim N, Ahmadiani A (2017). Mitochondrial dysfunction and biogenesis in neurodegenerative diseases: pathogenesis and treatment. CNS Neurosci Ther.

[46] Lin MT, Beal MF (2006). Mitochondrial dysfunction and oxidative stress in neurodegenerative diseases. Nature.

[47] Surma M, Anbarasu K, Dutta S, Olivera Perez LJ, Huang KC, Meyer JS (2023). Enhanced mitochondrial biogenesis promotes neuroprotection in human pluripotent stem cell derived retinal ganglion cells. Commun Biol.

[48] Greene L, Tischler A (1976). Establishment of a noradrenergic clonal line of rat adrenal pheochromocytoma cells which respond to nerve growth factor. Proc Natl Acad Sci USA..

[49] Yazdi IK, Taghipour N, Hmaidan S, Palomba R, Scaria S, Munoz A (2016). Antibody-mediated inhibition of Nogo-A signaling promotes neurite growth in PC-12 cells. J Tissue Eng.

[50] Luo S, Lan T, Liao W, Zhao M, Yang H (2012). Genistein inhibits Abeta(2)(5)(-)(3)(5) -induced neurotoxicity in PC12 cells via PKC signaling pathway. Neurochem Res.

[51] Iannetti EF, Smeitink JA, Beyrath J, Willems PH, Koopman WJ (2016). Multiplexed high-content analysis of mitochondrial morphofunction using live-cell microscopy. Nat Protoc.

[52] Lam J, Katti P, Biete M, Mungai M, AshShareef S, Neikirk K (2021). A universal approach to analyzing transmission electron microscopy with ImageJ. Cells.

[53] Zhang J, Liu X, Liang X, Lu Y, Zhu L, Fu R (2017). A novel ADOA-associated OPA1 mutation alters the mitochondrial function, membrane potential, ROS production and apoptosis. Sci Rep.

