# Supplementary material for: PGC-1a mediated mitochondrial biogenesis promotes recovery and survival of neuronal cells from cellular degeneration
Source: Cell Death Discov. 2024 Apr 17;10:180. doi: 10.1038/s41420-024-01953-0 (PMC11024166; doi:10.1038/s41420-024-01953-0)

**Original full length western blots**

Source data_Fig.4A_LC3I/II


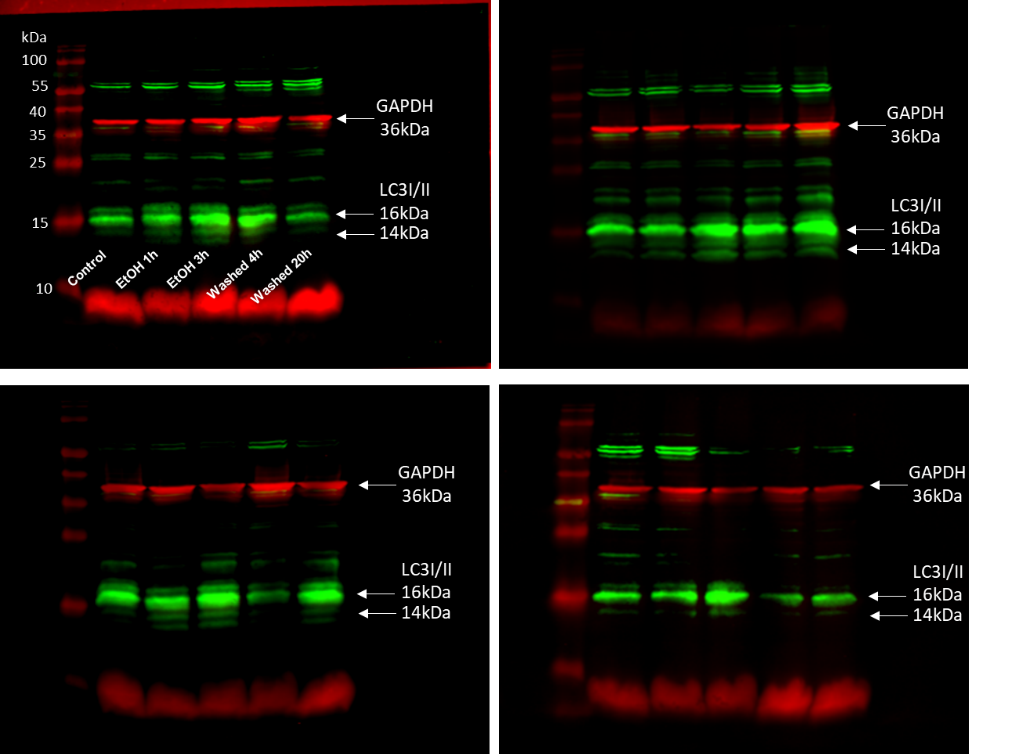


Source data_Fig.4A_p62


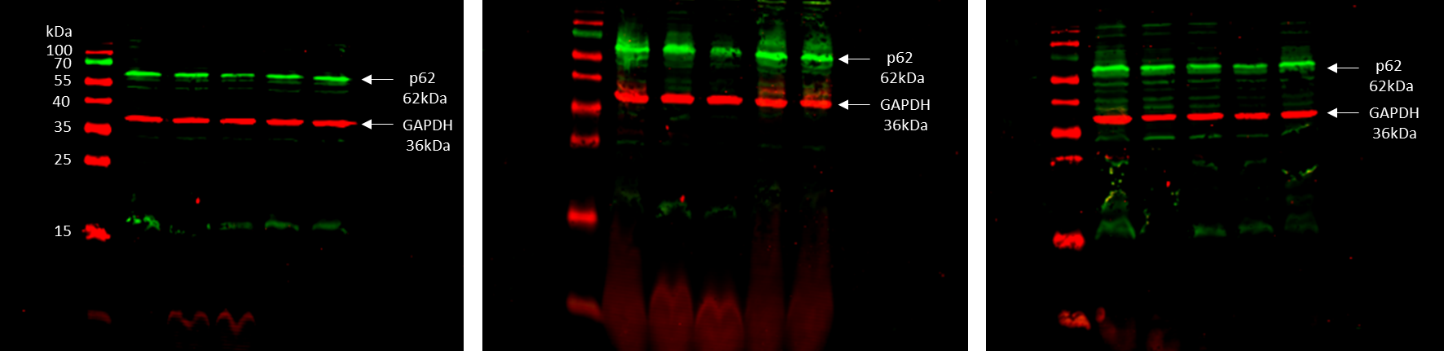


Source data_Fig.4A_Parkin

**
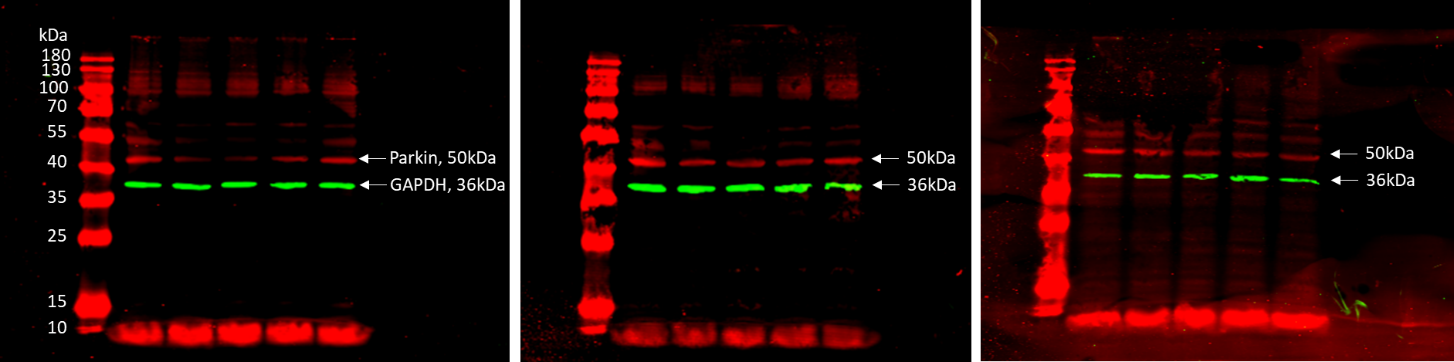
**

Source data_Fig.4A_PINK1

**
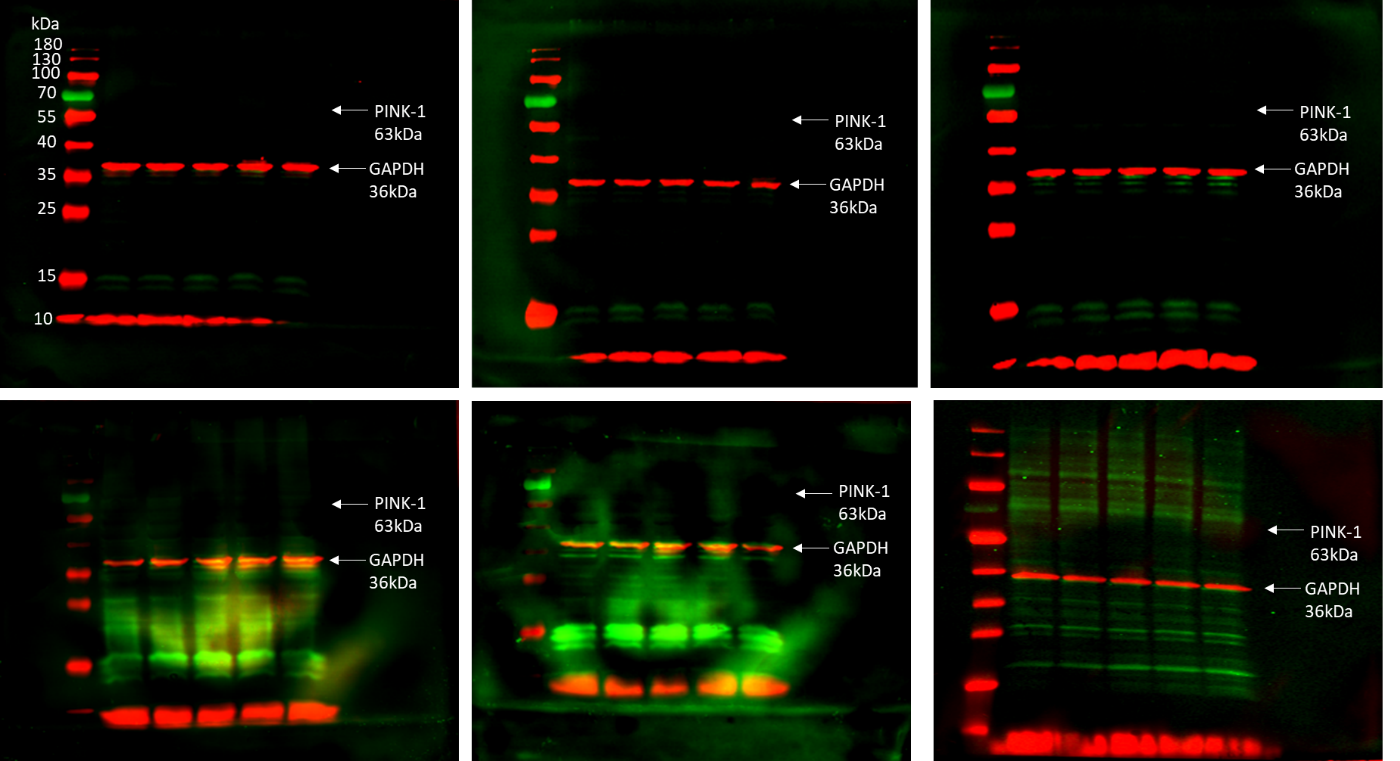
**

Source data_Fig.5E_Drp1

**
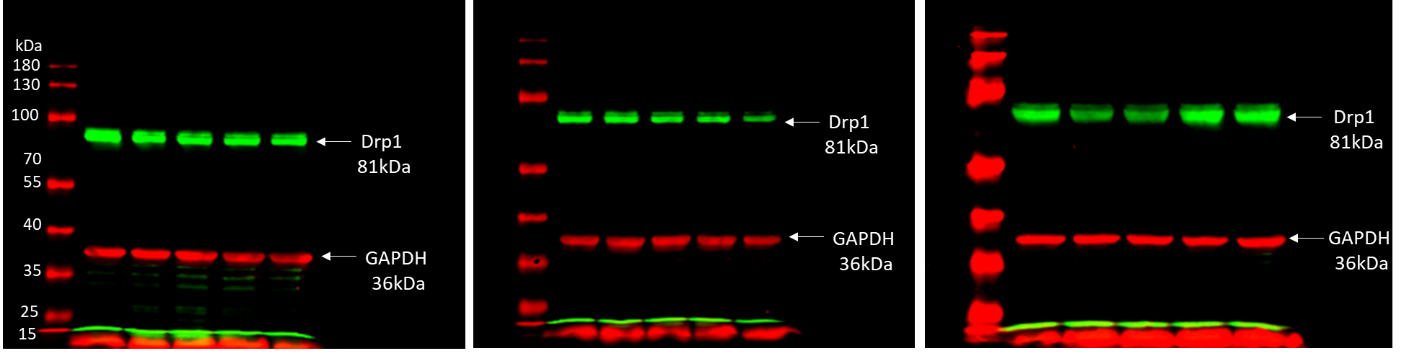
**

Source data_Fig.5E_p-Drp1(Ser616)

**
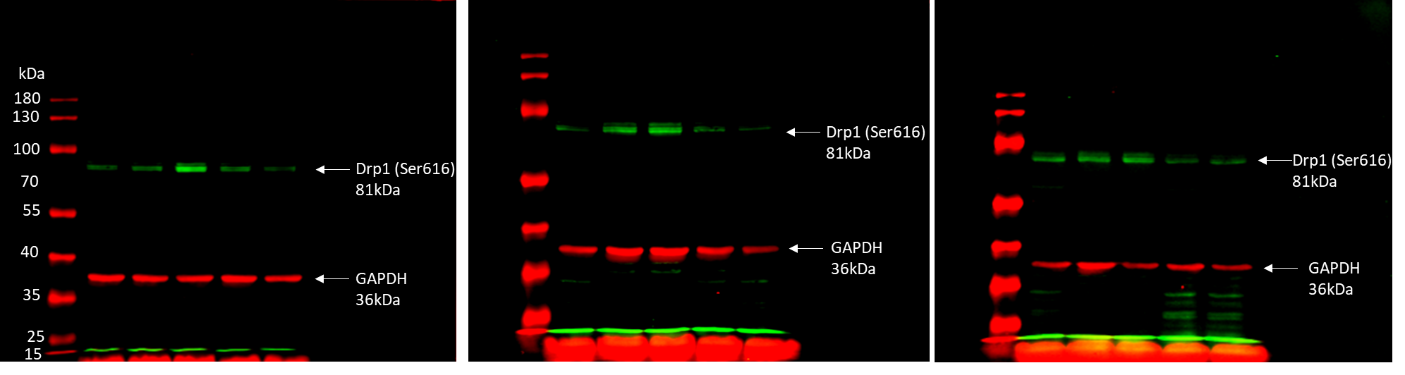
**

Source data_Fig.5E_p-Drp1(Ser637)


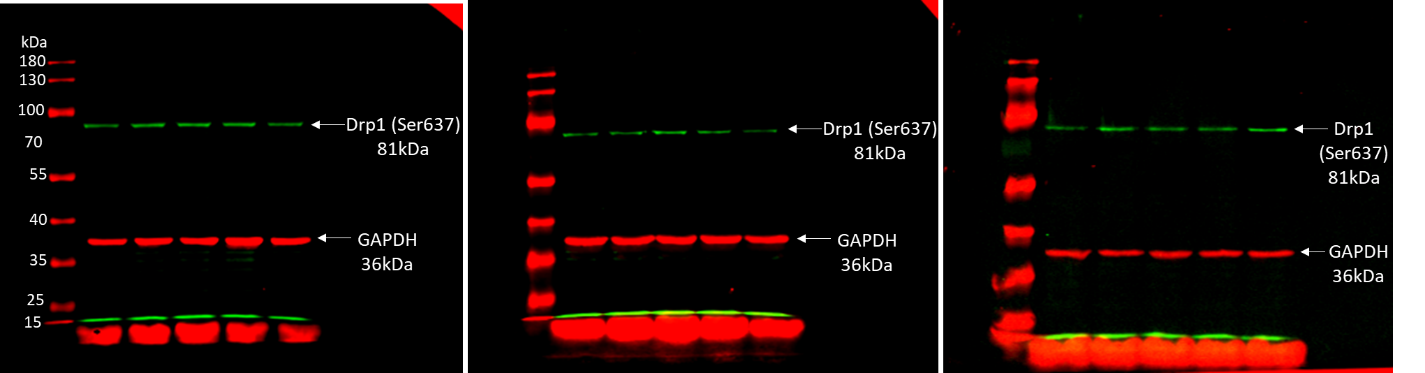


Source data_Fig.5E_OPA1

**
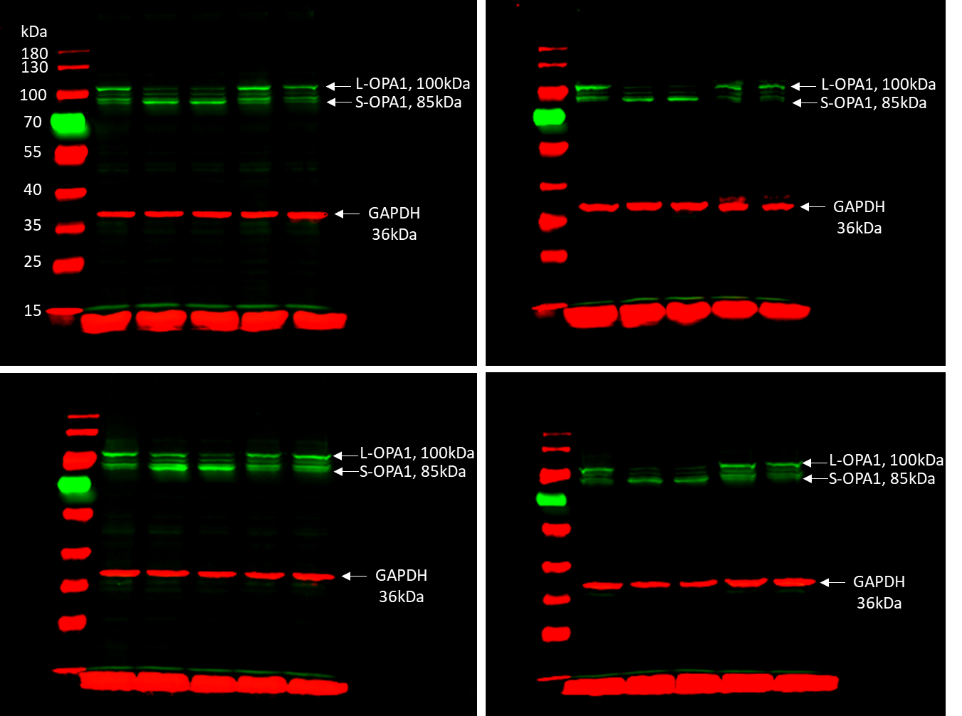
**

Source data_Fig.5E_MFN1

**
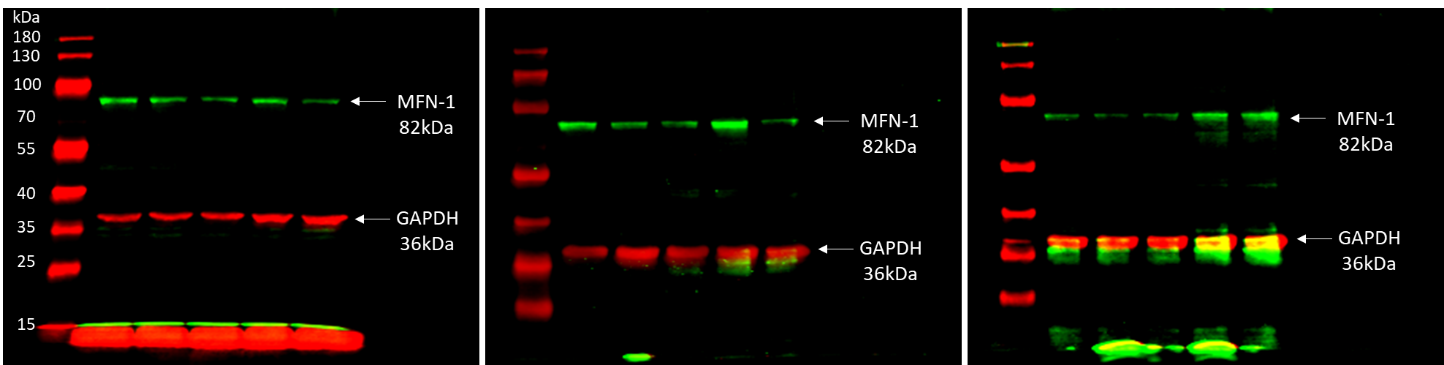
**

Source data_Fig.5E_MFN2

**
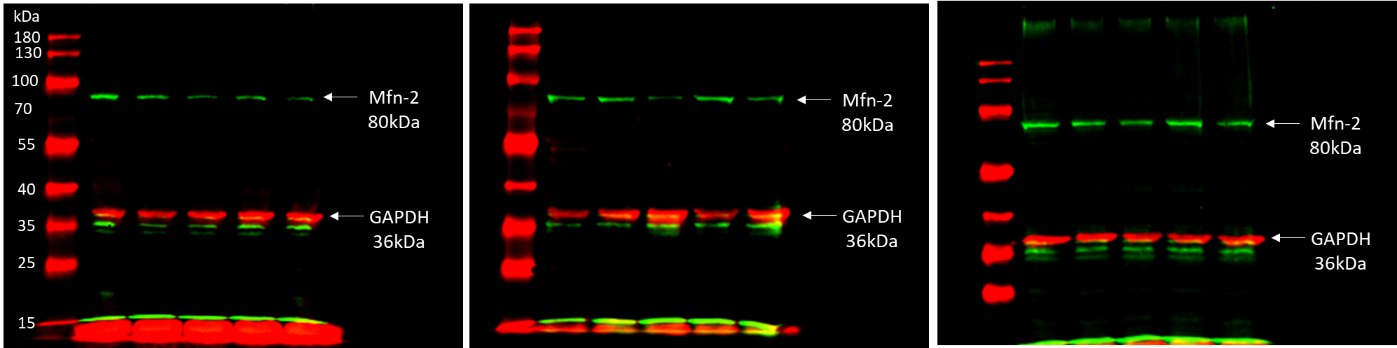
**

Source data_Fig.6D_PGC-1α


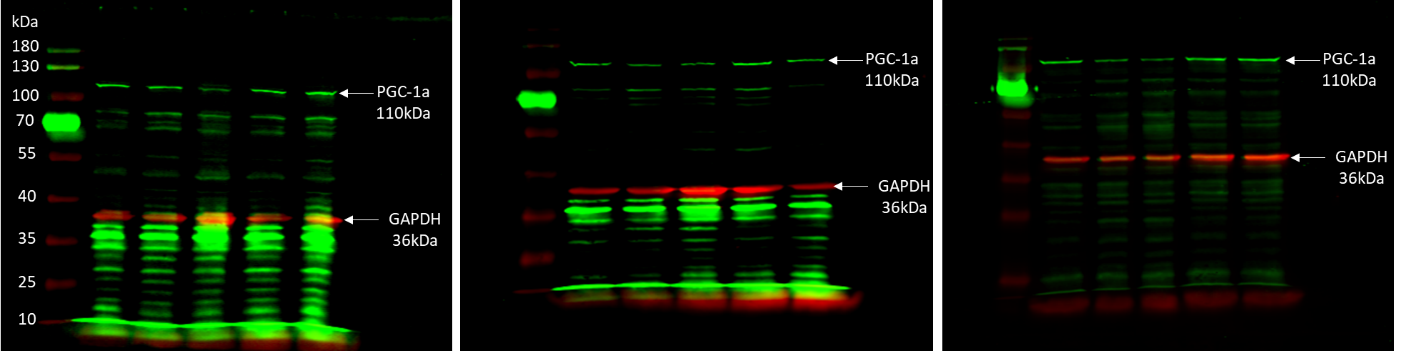


Source data_Fig.6D_AMPK-1α

**
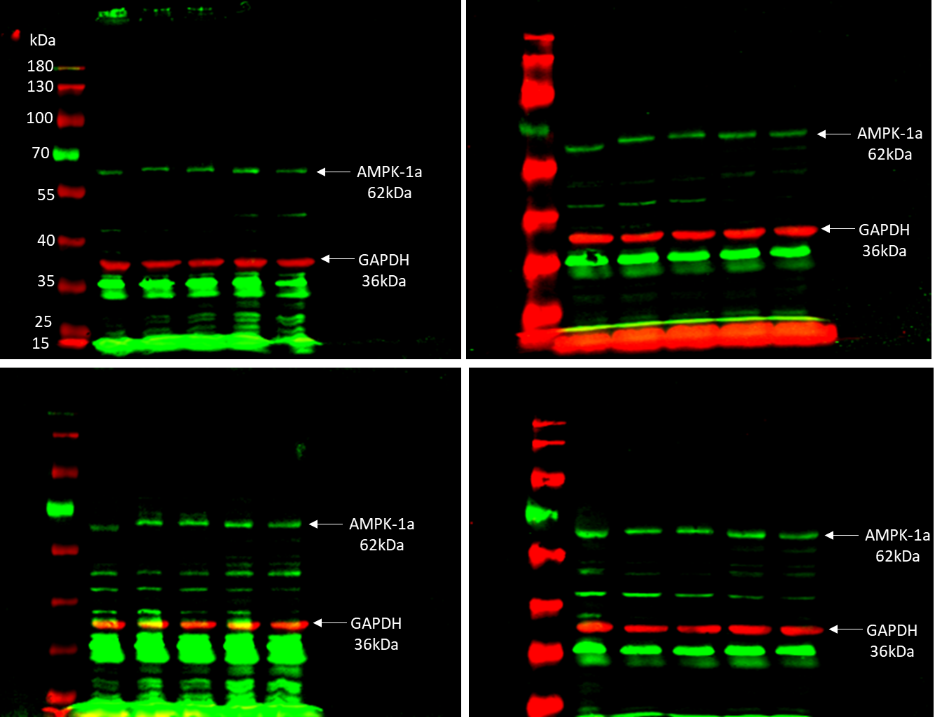
**

Source data_Fig.6D_p-AMPK-1α

**
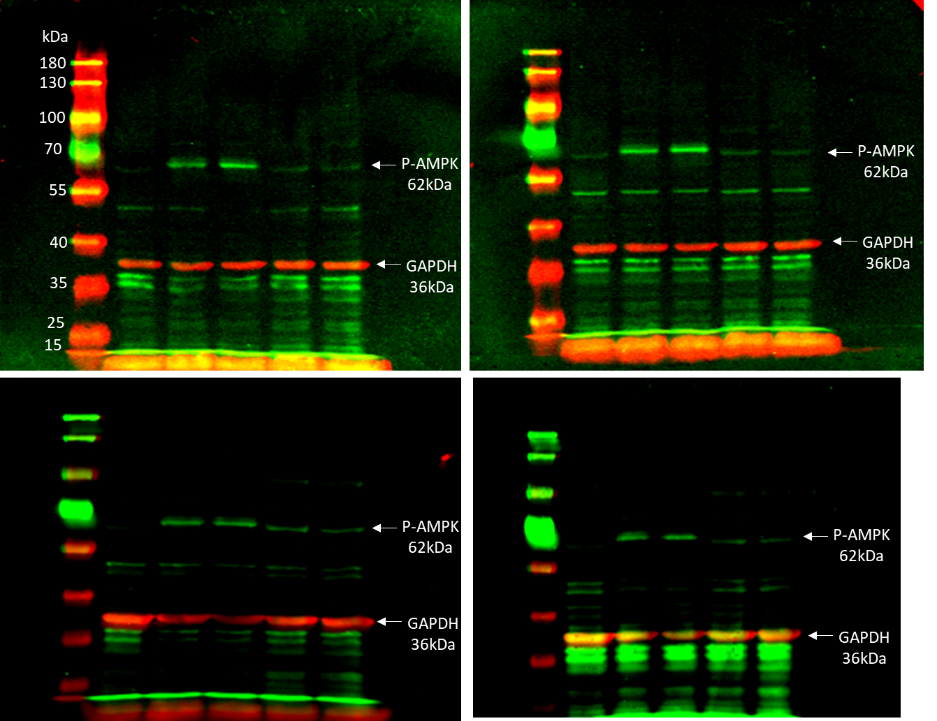
**

Source data_Fig.6D_SIRT1

**
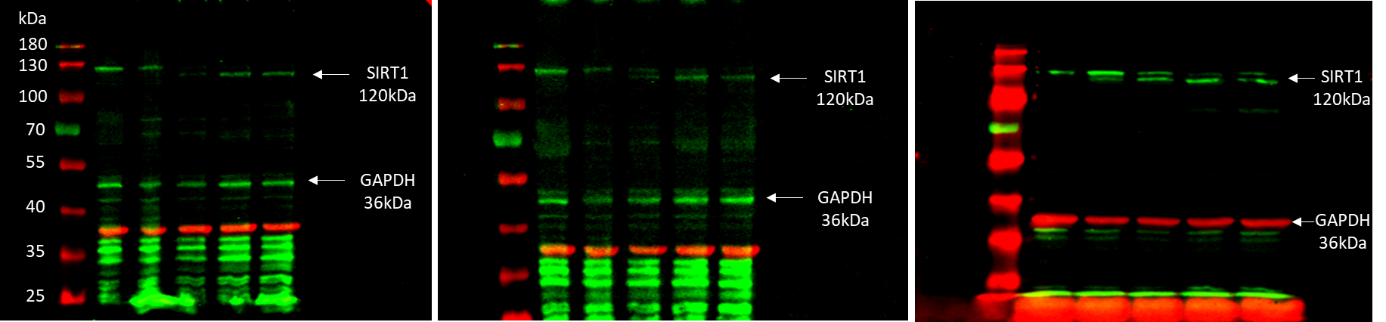
**

Source data_Supplemental Figure 1_LC3-I/II


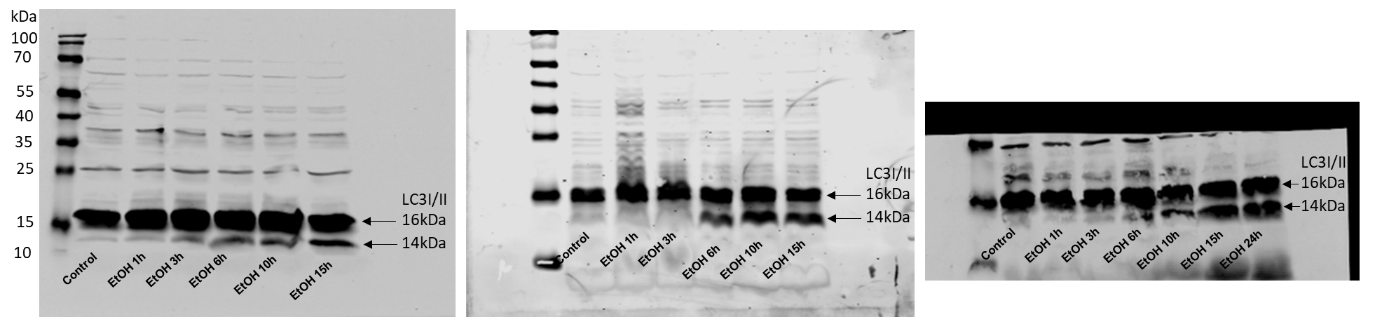

Supplement: Supplementary file 1 — Original full length western blots [file 41420_2024_1953_MOESM1_ESM.docx]
