# Supplementary material for: PGC-1a mediated mitochondrial biogenesis promotes recovery and survival of neuronal cells from cellular degeneration
Source: Cell Death Discov. 2024 Apr 17;10:180. doi: 10.1038/s41420-024-01953-0 (PMC11024166; doi:10.1038/s41420-024-01953-0)
Supplement: Supplementary file 2 — Supplementary information [file 41420_2024_1953_MOESM2_ESM.docx]

**PGC-1a mediated mitochondrial biogenesis promotes recovery and survival of neuronal cells from cellular degeneration**

**Wenting You ^1,2,3^,** **Kèvin Knoops ^4^, Tos Berendschot ^1^, Birke Benedikter ^1^, Carroll Webers ^1^, Chris Reutelingsperger ^2,^* and Theo Gorgels ^1,^***

^1^ University Eye Clinic Maastricht UMC+, Maastricht University Medical Center+, 6229 HX Maastricht, The Netherlands;

^2^ Department of Biochemistry, CARIM School for Cardiovascular Disease, Maastricht University, 6229 ER Maastricht, The Netherlands;

^3^ Department of Mental Health and Neuroscience, Maastricht University, 6229 ER Maastricht, The Netherlands;

^4^ The Microscopy CORE lab, Maastricht Multimodal Molecular Imaging Institute, Maastricht University, 6229 ER Maastricht, The Netherlands;

***** Correspondence: Theo G.M.F. Gorgels, [theo.gorgels@mumc.nl](mailto:theo.gorgels@mumc.nl); Tel.: +31-433871565.

Chris P.M. Reutelingsperger, [c.reutelingsperger@maastrichtuniversity.nl](mailto:c.reutelingsperger@maastrichtuniversity.nl); Tel: +31-433881533.

**Supplemental Table 1.**

Primer sequences used for qRT-PCR

Gene name Forward primer (5ˊ-3ˊ) Reverse primer (5ˊ-3ˊ)

Drp1 TGGAAAGAGCTCAGTGCTGG ACTCCATTTTCTTCTCCTGTTGT

OPA1 ATTTCGCTCCTGACCTGGAC GGTGTACCCGCAGTGAAGAA

MFN1 CACTTTTGCTCGACTGTGCC CTCGGGTGGAGAAACTGCTT

MFN2 ACCAGCTAGAAACGAGATGTCC GTGCTTGAGAGGGGAAGCAT

PGC-1α GCAGTCGCAACATGCTCAAG GGGAACCCTTGGGGTCATTT

AMPK-1α TAGCCGACTTCGGTCTTTCA CATAATTGGGCGAGCCACAG

SIRT1 ACCAGTAGCACTAATTCCAAGTTC TGGCATACTCGCCACCTAAC

GAPDH CTCTCTGCTCCTCCCTGTTC TACGGCCAAATCCGTTCACA

**Supplemental Figure 1**

**
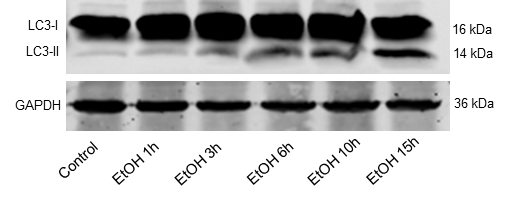
**

**Supplemental Figure 1: EtOH induced autophagy in neuronal PC12 cells.** Neuronal PC12 cells were exposure to treatment with or without ethanol (5%, vol/vol) for varying durations (1, 3, 6, 10, 15 h). The protein expression of LC3-I/II was assessed through western blot analysis. The results revealed that with prolonged treatment duration, there was an increased conversion of LC3-I to LC3-II, indicating an increased activation of autophagy. EtOH: ethanol.
